# Inactivation of the *sco2730/2731* copper chaperone–transporter system in *Streptomyces coelicolor* and its orthologs in *Streptomyces venezuelae*, together with chromosomal end deletion, greatly enhances secondary metabolism

**DOI:** 10.1186/s12934-026-03000-2

**Published:** 2026-04-06

**Authors:** Gemma Fernández-García, Paula Valdés-Chiara, Paula García-Cancela, Nathaly González-Quiñónez, Juan Serna-Diestro, Felipe Lombó, María Montes-Bayón, Ángel Manteca

**Affiliations:** 1 Department of Functional Biology, Microbiology Area, IUOPA and ISPA, Faculty of Medicine, Universidad de Oviedo, c/ Julian Claveria 6, 33006 Oviedo, Spain; 2 Department of Physical and Analytical Chemistry, Faculty of Chemistry and ISPA, Universidad de Oviedo, c/Julian Claveria 8, 33006 Oviedo, Spain

**Keywords:** *Streptomyces*, Secondary metabolism, Copper homeostasis, Chromosomal end loss, Silent biosynthetic pathways

## Abstract

**Background:**

Activating silent biosynthetic gene clusters (BGCs) in *Streptomyces* remains a major challenge in harnessing their vast secondary metabolic potential and requires diverse, complementary strategies, including bacterial co-cultivation, heterologous expression, chemical elicitation, modulation of gene expression, and the use of pleiotropic and pathway-specific genetic regulators, such as those influenced by cytosolic copper levels. Previous studies reported that, in *S. coelicolor*, disruption of the *sco2730/2731* copper chaperone–transporter system (Sc-M1 mutant) markedly enhances secondary metabolism. However, this activation is only partially reproduced by antisense knockdown constructs targeting *sco2730/sco2731* in *S. coelicolor* (Sc-M2 mutant) and other species (*S. venezuelae*, *S. albidoflavus*). This study investigates the basis of the strong activation observed in Sc-M1, with the aim of exploiting this mechanism for activating silent BGCs in *Streptomyces*.

**Results:**

Genomic analysis revealed that, in addition to *sco2730/2731* inactivation, the Sc-M1 mutant possesses a spontaneous deletion of both chromosomal ends. Construction of a *sco2730* knockout mutant (Δ*sco2730*, also affecting *sco2731*; Sc-M3 mutant) showed an effect on secondary metabolism comparable to that of the Sc-M2 mutant, and demonstrated that *sco2730* disruption increases chromosomal-end instability. Metabolomic analyses showed that inactivation of *sco2730/2731* (Sc-M3 mutant) or chromosomal-end deletion (Sc-M4 mutant) individually enhanced secondary metabolism. However, only the combination of Δ*sco2730* and chromosomal-end deletion (Sc-M5 mutant) approached the extensive metabolic activation observed in Sc-M1, affecting up to 60 secondary-metabolite adducts from 17 biosynthetic pathways. Similar synergistic effects were observed in *S. venezuelae*, where combined knockdown of the *sco2730/2731* orthologues and chromosomal-end deletion strongly modulated secondary metabolism, repressing chloramphenicol production while inducing pikromycin biosynthesis, a typically silent and difficult-to-activate *S. venezuelae* BGC.

**Conclusions:**

Simultaneous disruption of the *sco2730/31* copper chaperone–transporter system and chromosomal-end deletion synergistically enhance secondary metabolism production in *S. coelicolor* and *S. venezuelae*. This combined genetic manipulation provides a novel strategy for the challenging task of activating silent biosynthetic pathways and for potentially discovering new bioactive compounds across *Streptomyces* species.

**Supplementary Information:**

The online version contains supplementary material available at 10.1186/s12934-026-03000-2.

## Background

*Streptomyces* species are renowned for their ability to produce a vast array of secondary metabolites, including many clinically important antibiotics, antitumour agents, and other bioactive compounds [1, 2]. They represented the principal source of antimicrobials during the “Golden Age of Antibiotics” (1930–1960) [3, 4]. Drug discovery became increasingly challenging once the most common antibiotics had been identified. At the same time, microbial resistance to existing drugs has risen dramatically, rendering certain infections extremely difficult to treat. Despite extensive research efforts, no genuine alternative to screening natural microbial strains has emerged for the discovery of new antibiotic scaffolds and families. *Streptomyces* therefore remain the most promising source of novel antimicrobial compounds. A typical *Streptomyces* genome encodes approximately 30 biosynthetic gene clusters (BGCs) for secondary metabolites, yet only about four are usually expressed under standard laboratory conditions [5–8]. Activating these silent pathways is essential to unlock the full biosynthetic potential of actinomycetes and enhance the likelihood of discovering new bioactive compounds [8–11].

The activation of silent BGCs remains a major challenge and must be pursued through diverse and complementary strategies, including modifying growth conditions (the “one strain, many compounds” or OSMAC approach), bacterial co-cultivation, heterologous expression, promoter engineering, ribosome and RNA polymerase engineering, stress induction, chemical elicitation, precursor supplementation through metabolic engineering, the mutation or overexpression of regulatory genes (reviewed in [8, 10–12]), and alterations in oxidative metabolism, which have also been shown to exert a strong influence on secondary metabolism [13, 14]. The *Streptomyces* genus is characterised by a complex developmental cycle involving vegetative and reproductive mycelia that differentiate into spores [15, 16]. Hyphal differentiation is tightly regulated and closely linked to secondary metabolism in both solid (Petri dish) and liquid (flask or bioreactor) cultures [17–19]. Many regulators of hyphal differentiation also exert pleiotropic effects on secondary metabolism, and their mutation or overexpression has been shown to enhance secondary metabolite production in various *Streptomyces* species. Notable examples include the sensor kinase AbsA1 [20], the WhiB-like gene A (WblA) [21], AdpA [22, 23], and DasR [24, 25], among other well-characterised regulatory genes (reviewed in [26]). In recent years, a growing number of novel pleiotropic regulators have been described and evaluated for their ability to activate secondary metabolism. These include the nucleoid-associated protein Lsr2 [27], the XRE-family transcription factor Scr1 [28], the two-component systems AtcR/AtcK [29] and AbrC1/C2/C3 [30, 31], and the diguanylate cyclase CdgB [32]. Numerous pathway-specific transcriptional regulators from the TetR, GntR, SARP, LuxR, WhiB, MarR, and LysR families have also been reported to modulate secondary metabolism, some with broad pleiotropic effects, and others acting on a single pathway (reviewed in [33]). Further exploration of these regulatory mechanisms holds considerable promise for unlocking silent biosynthetic pathways in actinomycetes.

Building on the activation of secondary metabolism via pleiotropic regulators, it has become well established over the past decade that both extracellular and cytosolic copper play key roles in *Streptomyces* development, including aerial mycelium formation and sporulation, as well as antibiotic production [34–39]. Cytosolic copper levels are tightly regulated by copper chaperones and transporters whose gene expression is finely tuned [40]. Disruption of the *S. coelicolor sco2730* copper chaperone open reading frame (*sco2730::Tn5062* mutant, referred to in this study as Sc-M1) abolishes *sco2730* expression and reduces the expression of *sco2731*, which encodes a P-type ATPase copper exporter. This results in increased cytosolic copper levels and a substantial enhancement of secondary metabolism [41]. Twelve BGCs (43.3% of the 30 predicted clusters in *S. coelicolor*), including three previously silent pathways (coelimycin P1, dipeptide, and lantibiotic), were activated in this mutant [36, 41]. The strong conservation of the *sco2730/31* copper chaperone/transporter system across streptomycetes [41] makes it a promising target for the activation of secondary metabolism. In a previous study, we constructed an antisense mRNA against *sco2730/31* (referred to as the Sc-M2 mutant), which successfully activated secondary metabolism in three model strains (*S. coelicolor*, *S. venezuelae*, and *S. albidoflavus*), although to a lower degree, approximately half in the case of *S. coelicolor*, compared with the original Sc-M1 mutant [42].

The main objective of the present study was to understand and reproduce the massive activation observed in the Sc-M1 mutant, with the aim of applying this knowledge to the activation of silent biosynthetic gene clusters in *Streptomyces*. To this end, we investigated the differences between the Sc-M1 and Sc-M2 mutants and discovered that the Sc-M1 mutant exhibits a high rate of spontaneous chromosomal end deletion. The combination of *sco2730/2731* inactivation, or inactivation of their orthologues, with chromosomal end deletion leads to a strong activation of secondary metabolism in the model strains *S. coelicolor* and *S. venezuelae*.

## Methods

### Bacterial strains and culture conditions

All *Streptomyces* and *Escherichia coli* strains used in this study are listed in Additional File 1.

*Streptomyces coelicolor* M145 spores were harvested from SFM solid plates [43] after incubation at 30 °C for 7 days. Germination assays were performed on GYM agar (5 g/L glucose, 4 g/L yeast extract, 5 g/L malt extract, 0.5 g/L MgSO₄·7 H₂O, 20 g/L agar; supplemented after autoclaving with 0.5 g/L K₂HPO₄), overlaid with sterile cellophane and inoculated with 10⁷ spores from a fresh (non-frozen) suspension. Fermentations were carried out in 50 mL sucrose-free R5A medium [44] in 250 mL baffled flasks at 30 °C and 200 rpm, inoculated with 10⁷ spores/mL.

*Streptomyces venezuelae* NRRL B-65,442 spores were harvested from TBO plates (tomato purée, oatmeal, agar) [45] after 4 days at 30 °C. The pre-inoculum was prepared by cultivating 5 × 10⁶ fresh spores in 10 mL TSB (Scharlau) for 16 h at 30 °C and 220 rpm. Fermentations were conducted in 20 mL MYM medium supplemented with 2.1 g/L MOPS, inoculated with 2 mL of pre-inoculum and incubated at 30 °C and 220 rpm. After 8 h, ethanol was added to a final concentration of 6% (v/v) to induce chloramphenicol production [46].

*E. coli* strains were grown in 2×TY at 37 °C.

Antibiotics were added at the following concentrations: apramycin (100 µg/mL for *E. coli*, 25 µg/mL for *S. coelicolor*), hygromycin (100 µg/mL for *E. coli*, 200 µg/mL for *S. coelicolor*), kanamycin (50 µg/mL), chloramphenicol (25 µg/mL), and nalidixic acid (25 µg/mL) (all from Invitrogen, USA).

### Chromosomal end deletions in the Sc-M1 mutant and identification of the closest 5′ and 3′ regions in model *Streptomyces* strains

**Fig. 1:**
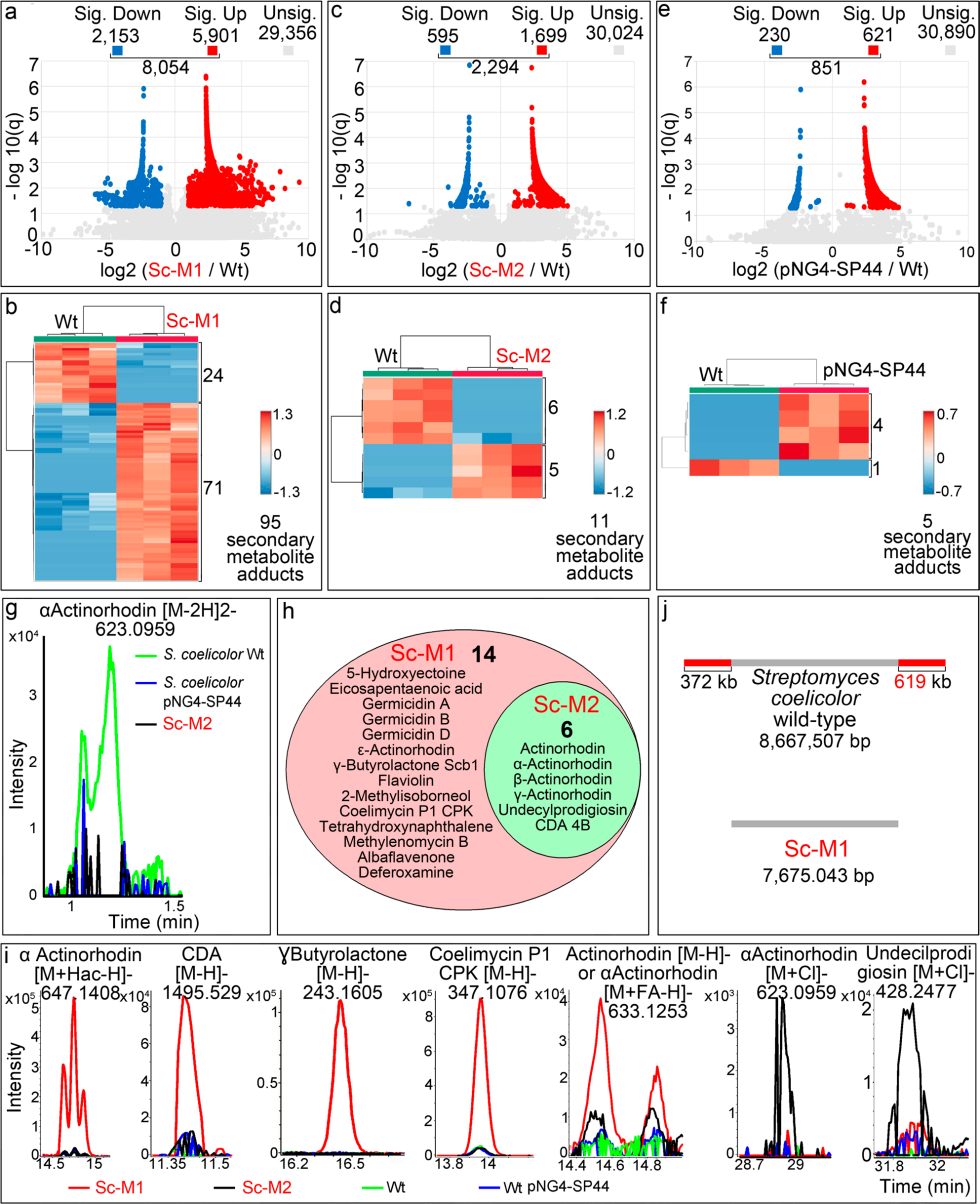
Differences in the metabolome between the *sco2730::Tn5062* Sc-M1 mutant, the Sc-M2 mutant, and the *S. coelicolor* wild-type strain grown on R5A medium at the time point of maximum antibiotic production (145 h). **a**, **c**, **e** Volcano plots showing the number of metabolites with significant differences (q-value ≤ 0.05 and log₂ (mutant / *S. coelicolor* wild-type) ≥ +/-1) and non-significant differences. pNG4-SP44 corresponds to the *S. coelicolor* control strain harbouring the empty pNG4-SP44 expression plasmid. **b**, **d**, **f** Heat maps illustrating the abundance of secondary-metabolite adducts with significant differences across the three biological replicates. **g**, **i** Extracted ion chromatograms showing representative secondary-metabolite adducts with significantly altered abundance between the mutants and the wild-type strain. Colours indicate: red, Sc-M1; black, Sc-M2; green, *S. coelicolor* wild type; blue, *S. coelicolor* harbouring the empty pNG4-SP44 plasmid. **h** Venn diagram showing secondary metabolites with adducts differentially abundant between the Sc-M1 and Sc-M2 mutants relative to the wild type. **j** Schematic illustrating chromosomal end loss in the Sc-M1 mutant

Chromosomal end deletion in the Sc-M1 mutant was identified by next-generation sequencing (NGS) of genomic DNA, subcontracted to StabVida (Portugal). Two terminal deletions were detected at positions 372,937 bp and 8,047,980 bp (Fig. 1j). To determine the closest conserved regions adjacent to these deleted ends, we queried the StrepDB database (http://strepdb.streptomyces.org.uk/) and compared syntenic conservation across six model *Streptomyces* species: *S. coelicolor*, *S. lividans*, *S. avermitilis*, *S. venezuelae*, *S. griseus* and *S. clavuligerus* using the BLASTn tool. We selected 2,000 bp regions surrounding the *S. coelicolor* coordinates 372,937 bp and 8,047,980 bp that showed at least 70% nucleotide identity across these species.

### Construction of the Sc-M1–M5 mutants in *S. coelicolor*

Mutants Sc-M1 (*sco2730::Tn5062*) and Sc-M2 (antisense knockdown of *sco2730/31*) were described previously [36, 42].

The Δ*sco2730* mutant (Sc-M3) was generated by replacing *sco2730* with the apramycin resistance gene. A deletion construct containing the apramycin resistance cassette, including the promoter and T4 transcriptional terminator of the *Tn5062* transposon [47], flanked by 1.4 kb upstream and downstream *sco2730*, was synthesised (GeneCust, France) and cloned into pCR-Blunt II-TOPO (Thermo Fisher), generating TOPO-*sco2730*up-*apra*-*sco2730*down. The *oriT* fragment amplified from pNG3 [48] using primers *oriTFa*/*oriTRa* (Additional File 2) was cloned into pCR-Blunt II-TOPO generating TOPO-*oriT*. After sequence verification (Sanger sequencing; primers *M13F*/*M13R*), the *oriT* fragment was excised with *Pci*I/*Hpa*I and inserted into TOPO-*sco2730*up-*apra*-*sco2730*down. The final construct was confirmed by Sanger sequencing (*oriTPciI*,* oriTFb*,* oriTRb*,* oriT2730*) (Additional File 2) and introduced into *S. coelicolor* via conjugation using the protocol of Kieser et al. [43]. Sc-M3 mutants grew on apramycin but not kanamycin. Correct *sco2730* deletion was confirmed by PCR (primers *Tn2730F/R*) (Additional File 2).

**Fig. 2:**
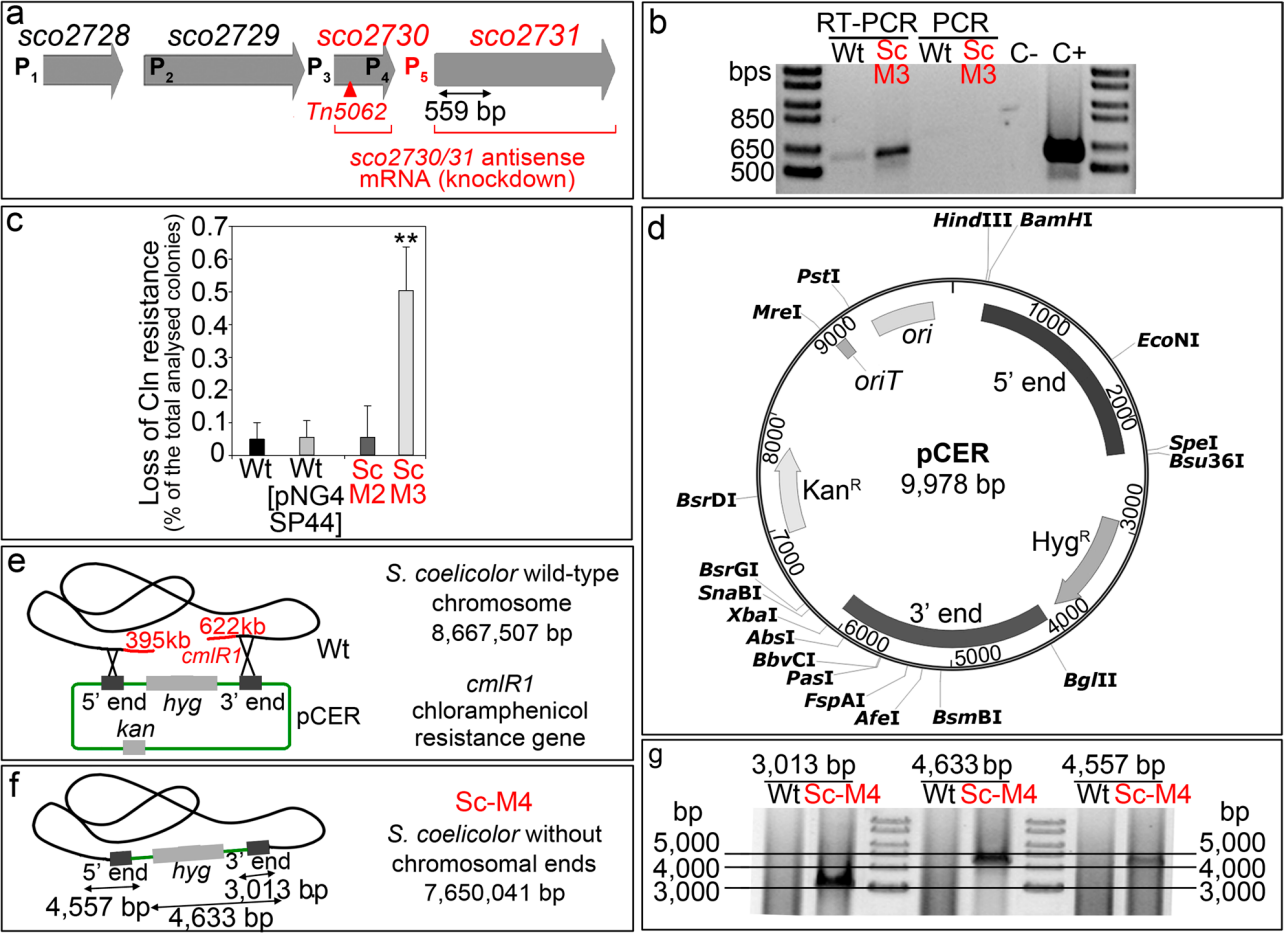
Complexity of the*sco2730–2731*genetic region, chromosomal instability in the Δ*sco2730*knockout (Sc-M3 mutant), and generation of the chromosomal end-deletion mutant (Sc-M4) using the pCER plasmid. **a ***sco2730/31* genetic region. The insertion site of the *Tn5062* transposon is indicated by a triangle. The four promoters previously described by González-Quiñonez et al. [36] are shown in black, and the newly identified promoter is shown in red. The region targeted by the antisense mRNA is outlined in red, and the 559 bp fragment amplified by RT-PCR is indicated by a double-headed arrow. **b** RT-PCR analysis of the *sco2731* ORF in the Sc-M3 mutant and the wild-type strain. The absence of amplification by conventional PCR from RNA templates confirms the absence of contaminating DNA. Positive control: conventional PCR using chromosomal DNA as template. Negative control: RT-PCR using water instead of RNA as template. **c** Percentage of chromosomal end loss (estimated as loss of chloramphenicol resistance) in Sc-M2, Sc-M3, and *S. coelicolor* strains with and without the empty pNG4 plasmid. **d** Map of the pCER plasmid containing the 5′ and 3′ *S. coelicolor* chromosomal end regions in the correct orientation to facilitate chromosomal end deletion by homologous recombination. **e** Schematic representation of chromosomal end loss by recombination. *cmlR1* indicates the chloramphenicol resistance gene located at the 3′ end of the chromosome. **f** Diagram of the circular chromosome in the Sc-M4 mutant. **g** Confirmation of chromosomal end loss and circularisation by PCR. The amplified DNA fragments are illustrated in (**f**). Asterisks indicate significant differences compared with the wild type: *p < 0.05, **p < 0.01, ***p < 0.001. Error bars represent standard deviations (SDs). Uncropped versions of the gels are shown in Additional File 7

*S. coelicolor* lacking chromosomal ends (Sc-M4) was constructed using pCER plasmid (Fig. 2d) which can only integrate into *Streptomyces* via homologous recombination, as it is unable to replicate in this genus. This vector was generated from pCR-Blunt II-TOPO, first by cloning of the hygromycin resistance cassette (amplified from pMS82 [48] with *hygroF/R*) (Additional File 2). The 5′ and 3′ chromosomal ends (amplified from wild-type genomic DNA using *5primaF/R* and *3primaF/R*) (Additional File 2) and the *oriT* fragment (excised from TOPO-*oriT* with *EcoR*V/*Zra*I) were subsequently inserted. Assembly was carried out using the appropriate restriction enzymes (*BamH*I/*Spe*I for the 5′ fragment, *EcoR*V/*Not*I for the 3′ fragment, *Dra*I for the *oriT* fragment) and T4 DNA ligase (Thermo Fisher). All fragments were sequence-verified by Sanger sequencing using primers *M13F*/*R*, *OriTFb/Rb*, and *TOPOHindIII/5pSeI* (Additional File 2). The final plasmid was introduced into *E. coli* ET12567/pUZ8002 and conjugated into *S. coelicolor*, generating Sc-M4 mutant. Correct mutants were identified by antibiotic selection and PCR (*110V3F1/R1*, *110V3F1/hygroR*, and *hygroF/110V3R2*) (Additional File 2).

The Sc-M5 mutant was generated by introducing pCER into Sc-M3, thereby combining Δ*sco2730* with chromosomal end deletion. Confirmation was performed by antibiotic selection and PCR (*110V3F1/110V3R1*, *110V3F1/hygroR*, *hygroF/110V3R2* primers) (Additional File 2).

### Construction of the Sv-M1–M3 mutants in *S. venezuelae*

Sv-M1 (antisense *sco2730/31* RNA overexpression) was generated by cloning the *sco2730/31* antisense mRNA from pNG4-SP44 [42] into pRASK-SP44 via restriction cloning using *EcoR*V/*Nde*I and T4 DNA ligase (Thermo Fisher), and transferring the construct by conjugation [43] to *S. venezuelae*. Correct ϕC31 integration was confirmed by PCR using primers *sven3566F* and s*co3798intR* (Additional File 2).

Sv-M2 (mutant lacking chromosomal ends) was generated using pCER as described for *S. coelicolor*. Mutants were validated by antibiotic selection and PCR (*circu5F/R* and *circu3F/R* primers) (Additional File 2).

Sv-M3 mutant, which combined chromosomal end deletion with antisense *sco2730/31* RNA overexpression, was generated by introducing pCER into Sv-M1 by conjugation. Chromosomal end deletion was confirmed by PCR (*circu5F/R* and *circu3F/R* primers) (Additional File 2).

### Protein quantification

Protein concentration was measured by the Bradford assay [49] using BSA (Sigma-Aldrich) as a standard. Cell extracts were prepared by mixing culture aliquots with an equal volume of 1 M NaOH, boiling for 5 min, and removing debris by centrifugation at 7,740 × g.

### Quantification of spore germination

Germination assays were performed as previously described [36] on GYM agar [50] covered with cellophane and inoculated with 10⁷ spores. After 8 h at 30 °C, cellophane squares were cut and transferred onto coverslips, stained with SYTO9 and propidium iodide (LIVE/DEAD BacLight Kit, Invitrogen L1-3152), and visualised using a Leica TCS-SP8 confocal microscope (488 and 568 nm excitation; 530 nm and 640 nm emission) [17]. Germination was quantified for at least 100 spores per replicate using ImageJ/Fiji [51]. Three biological replicates were analysed. Data normality was assessed using the Shapiro–Wilk test and variance homogeneity by Levene’s test. Statistical significance was determined by one-sided *t*-tests (*p* < 0.05, < 0.01, < 0.001).

### Quantification of chromosomal end-loss rate in *S. coelicolor*

Chromosomal end loss was quantified using the classical chloramphenicol-resistance assay [52]. *S. coelicolor* is naturally resistant to chloramphenicol, with the resistance determinant located at the 3′ chromosomal end; loss of resistance indicates end deletion and subsequent circularisation [52]. Dilutions of spores were plated on GYM agar. Colonies were replica-plated using a sterile velvet replicator onto non-selective and chloramphenicol-amended plates (8 µg/mL). Colonies failing to grow on chloramphenicol were scored as chromosomal-end deletion events. Three biological replicates were performed. Data normality and variance homogeneity were assessed as above. Differences in loss rates were evaluated using one-sided *t*-tests (*p* < 0.05, < 0.01, < 0.001).

### DNA and RNA extraction

Genomic DNA was isolated following standard methods [43]. Total RNA was extracted using Direct-zol™ RNA columns (Zymo-Spin™; Zymo Research, Irvine, CA, USA) and treated with TURBO™ DNase (Thermo Fisher Scientific, Waltham, MA, USA). The quantity and integrity of RNA samples were assessed using a NanoDrop 2000 spectrophotometer (Thermo Fisher Scientific) and a 2100 Bioanalyzer (Agilent Technologies, Santa Clara, CA, USA).

### Reverse transcription-PCR (RT-PCR)

RT-PCR analysis was performed using the SuperScript one-step RT-PCR system with Platinum Taq DNA polymerase (Invitrogen), with 200 ng of total RNA as template. Chromosomal DNA served as a positive control, and negative controls contained no template. Oligonucleotides *P5F*/*P5R* were used (Additional File 2). RT-PCR conditions were: first-strand cDNA synthesis at 60 °C for 30 min, followed by denaturation at 94 °C for 2 min; PCR amplification for 40 cycles at 94 °C for 15 s, 60 °C for 30 s (annealing), and 68 °C for 40 s (extension), followed by a final extension at 68 °C for 5 min. Conventional PCR was performed under the same conditions to confirm the absence of DNA contamination in RNA templates.

### Quantification of copper accumulation in single spores

Copper accumulation in individual spores was analysed as previously described [36]. Spores were collected from 7-day SFM cultures supplemented with 80 µM CuSO₄. A suspension of 15–20 mL of fresh spores (non-frozen) in distilled water was obtained from sporulated solid cultures. The suspension was filtered through sterile pipette tips filled with absorbent cotton to remove mycelial fragments, then centrifuged at 8,500 rpm for 10 min and the supernatant discarded. The pellet was washed three times with 10 mL washing buffer (10 mM Tris-HCl, pH 7.5; 1 mM EDTA) and finally resuspended in 10 mL phosphate-free TBS. Aliquots of 3–5 mL were further concentrated to 1 mL in sterile distilled water, achieving a final spore concentration of 10⁷–10⁸ spores/mL.

Copper content in single spores was measured by single-cell ICP-MS. Highly diluted spore suspensions (10⁵ spores/mL [36]) were introduced to prevent multiple cells entering the plasma within the same integration time. Samples were introduced into a triple-quadrupole ICP-MS (Thermo iCAP-TQ, Thermo Fisher Scientific, Bremen, Germany) employing an autosampler ASX-560 (Teledyne CETAC Technologies, Omaha, Nebraska, USA). The ICP-MS was operated in the single quad mode using helium as collision gas and was equipped with a Micro Mist nebulizer and a cyclonic spray chamber (both from ESI, Mainz, Germany). Data were acquired in time-resolved mode with 500 µs dwell time and 1 min per run. Differences in copper content per spore among Sc-M1–M5 mutants relative to the wild-type were analysed using the non-parametric Mann–Whitney U test, as the data did not follow a normal distribution, with p-values < 0.05, < 0.01, and < 0.001 considered significant.

### Compound extraction and HPLC-MS metabolome analysis

Compound extraction was performed as described previously [42]. Three biological replicates were collected at maximum secondary metabolite production (145 h for *S. coelicolor*, 48 h for *S. venezuelae*). Cells and supernatants from 20 mL cultures were separated by centrifugation at 10,000 rpm for 10 min. For *S. coelicolor*, both cells and supernatants were processed, whereas for *S. venezuelae*, only supernatants were processed. Supernatants were extracted with 0.56 volumes of ethyl acetate, vortexed three times for 2 min, and centrifuged at 10,000 rpm for 5 min. Cell pellets were extracted first with acetone (sonication: 2 min on, 2 min off; three cycles) and then with ethyl acetate, following the same vortexing and centrifugation protocol. Supernatants were combined and vacuum-dried using rotary evaporation (RV 10 Digital, IKA^®^-Werke GmbH & Co. KG, Germany). Dry extracts were stored at − 20 °C, resuspended in 4 mL methanol, transferred to 2 mL tubes, dried in a SpeedVac, and finally dissolved in 400 µL methanol: DMSO (1:1, v/v). Five µL of each sample were analysed by LC-HRESI-MS using a Dionex Ultimate 3000 UPLC system (Thermo Scientific, Waltham, MA, USA) coupled to an ESI-UHR-Qq-TOF Impact II spectrometer (Bruker, Billerica, MA, USA), in negative ion mode (m/z 40–2000 Da) on a Zorbax^®^ Eclipse Plus C18 column (50 × 2.1 mm, 1.8 μm; Agilent Technologies, Santa Clara, CA, USA).

*S. coelicolor* metabolites were eluted at 0.25 mL/min using a gradient of 0.1% formic acid in water (A) and 0.1% formic acid in acetonitrile (B): 0–10% B over 1 min, 10–35% B over 3 min, hold 35% for 1 min, 35–100% B over 3 min, hold 100% for 2 min, then return to 10% B over 1 min and hold for 4 min. *S. venezuelae* metabolites used the same flow rate and mobile phases with a gradient: 5% B at 0 min (hold to 3 min), 5–55% B to 28 min, 55–100% B to 41 min, hold 100% to 43 min. Chloramphenicol (98%, Acros Organics) was used as a standard to study the homogeneity between replicates and as a control for the production of metabolites from secondary metabolism in *S. venezuelae*.

### Bioinformatic analysis of the metabolome

Raw data were processed using DataAnalysis v4.3 (Bruker, Billerica, MA, USA) and calibrated with internal standards (sodium, formate acetone). For *S. coelicolor*, the R5A medium MOPS signal was used to verify calibration accuracy (average deviation 0.0001 Da). For *S. venezuelae*, chloramphenicol signal deviation after calibration was also 0.0001 Da. Chromatograms were further processed in MZmine 2 [53] using: centroid mass detection (noise level 2.0E2), ADAP chromatogram builder (minimum group 10, group intensity threshold 2.0E2, minimum highest intensity 5.0E2, m/z tolerance 0.001), smoothing (filter width 9), chromatogram deconvolution (local minimum search, threshold 90%, min RT range 0.05, min relative height 1%, min absolute height 5.0E2, min ratio peak top/edge 2, peak duration 0.03–3 min, m/z centre median), isotope grouping (m/z tolerance 0.001, RT tolerance 0.1, max charge 2, monoisotopic shape), representative isotope selection (most intense), join aligner (m/z tolerance 0.001, weight 20; RT tolerance 0.1, weight 20; isotope pattern m/z tolerance 0.001, min intensity 5.0E2, min score 50%), duplicate peak filtering (single feature, m/z tolerance 0.001, RT tolerance 0.1).

Processed data were exported to MetaboAnalyst 5.0 [54] for statistical analysis (missing values imputed as 1/5 minimum positive value, log10-transformed, pareto-scaled). Differences were considered significant at q ≤ 0.05. Secondary metabolites of *S. coelicolor* M145 and *S. venezuelae* NRRL B-65,442 were obtained from Nett et al. [41] and Lee et al. [6]. Molecular formulas and monoisotopic masses were obtained from PubChem (accessed 30 June 2025), and exact masses of ions and adducts were determined following Huang et al. [55]. Mass tolerance for metabolite identification was 0.001 Da; retention time tolerance for distinguishing isomers was 0.1 min.

## Results

### The *sco2730::Tn5062* Sc-M1 mutant has a broader impact on secondary metabolite production than the *sco2730/31* knockdown mutant (Sc-M2)

In a recent study we demonstrated that the *S. coelicolor sco2730/31* knockdown Sc-M2 mutant displays only a small fraction of the secondary metabolic activation observed in the *sco2730::Tn5062* Sc-M1 mutant [42]. In the present work, we have confirmed and extended these observations. The Sc-M1 mutant exhibits a pronounced alteration of its metabolome compared with the *S. coelicolor* wild-type strain, with 8,054 compounds detected (Fig. 1a), including 95 secondary-metabolite adducts, the majority of which are upregulated (red colour in Fig. 1b). This activation of secondary metabolism is substantially greater than that observed in the Sc-M2 mutant, in which only 2,294 compounds were altered (Fig. 1c), including 11 secondary-metabolite adducts, approximately eightfold fewer than in Sc-M1 (Fig. 1d) [42]. All secondary-metabolite adducts showing significant differences in the Sc-M1 and Sc-M2 mutants relative to the *S. coelicolor* wild-type strain are listed in Additional File 3.

The control strain carrying the empty plasmid pNG4-SP44 [42], the expression vector used to overexpress the *sco2730/31* antisense RNA in the Sc-M2 mutant, showed only a minor alteration in its metabolome (Fig. 1e), with five secondary-metabolite adducts differing in abundance relative to the wild-type strain (Fig. 1f). These changes were of markedly lower magnitude than those observed in either mutant; note that the colour scale in the control heatmap (–0.7 to 0.7) spans approximately half the range used for Sc-M1 and M2 (compare Figs. 1b, d, f). The only adduct showing significant variation relative to the wild type and shared by both Sc-M2 and the empty-vector control strain was αActinorhodin [M-2 H]2- (623.0959 m/z), whose abundance was markedly reduced in both Sc-M2 and the wild-type strain harbouring pNG4-SP44 (Fig. 1g). This reduction therefore appears to result from the presence of the plasmid. In contrast, the remaining differences in secondary metabolite adduct abundance observed in Sc-M2 relative to the *S. coelicolor* wild type can be attributed to the *sco2730/31* antisense RNA rather than to the expression vector itself.

When comparing secondary metabolites whose adducts show statistically significant differences in abundance between Sc-M1 and Sc-M2 mutants relative to the *S. coelicolor* wild type (q-value ≤ 0.05 and log₂ (mutant / *S. coelicolor* wild-type) ≥ +/-1), it becomes evident that Sc-M2 exhibits only a fraction of the secondary metabolic activation observed in Sc-M1 (Fig. 1h). Examples of secondary-metabolite adducts displaying significantly altered abundance between the mutants and the wild-type strain are shown in the chromatograms in Fig. 1i (chromatograms for all adducts displaying significant differences are presented in Additional File 5). The vast majority of these adducts are increased in both Sc-M1 (red trace, Fig. 1i) and Sc-M2 (black trace, Fig. 1i) compared with the *S. coelicolor* wild type carrying the empty expression plasmid (blue trace, Fig. 1i) or without a plasmid (green trace, Fig. 1i). Understanding the basis for the pronounced differences observed between the Sc-M1 and Sc-M2 mutants constitutes the main objective of this study, as explored in the following sections.

### The *sco2730::Tn5062* Sc-M1 mutant lacks chromosomal ends

To further investigate the differences between the Sc-M1 and Sc-M2 metabolomes, we sequenced the chromosome of the Sc-M1 mutant. In addition to the transposon insertion that inactivates *sco2730* and downregulates *sco2731* expression [36], we found that the mutant lacked 372 kb and 619 kb at its chromosomal termini (Fig. 1j). This finding raised the question of whether the activation of secondary metabolism in the Sc-M1 mutant depends on *sco2730/31* inactivation, chromosomal end deletion, or a combination of both. To address this question, we constructed three additional *S. coelicolor* mutants: a *sco2730* knockout mutant (Δ*sco2730*; Sc-M3); an *S. coelicolor* strain lacking chromosomal ends (Sc-M4); and a Δ*sco2730* mutant also lacking chromosomal ends (Sc-M5), thus mimicking the original Sc-M1 mutant. These mutants are described in the following sections.

### Transcription of the *sco2730–2731* genetic region is complex and is regulated by at least five distinct promoters

We attempted to construct an additional mutant, the *sco2731* knockout (Δ*sco2731*), but repeated efforts were unsuccessful, suggesting that the *sco2731*-encoded P-type ATPase copper exporter may be essential for cell viability. This finding appears to contradict the successful generation of the Δ*sco2730* mutant (Sc-M3), which in principle should also disrupt *sco2731*, because the four promoters described in this region are located upstream of the *sco2730* stop codon [36] (labelled as P1-P4 in Fig. 2a). Consequently, transcription from all four promoters should therefore be blocked by the strong T4 transcriptional terminator included downstream of the apramycin resistance gene used to replace *sco2730* (see Methods). To address this apparent contradiction, we analysed *sco2731* transcription in the Sc-M3 mutant by reverse transcription PCR (RT-PCR), amplifying a 559 bp region of the *sco2731* mRNA (Fig. 2b), which revealed the presence of at least a fifth promoter located downstream of *sco2730*, controlling *sco2731* expression in the Sc-M3 mutant and further contributing to the complex transcriptional regulation of *sco2730–2731*. Such regulatory complexity underlies the differences observed among the five mutants generated (described below), as each affects these promoters in distinct ways.

### The Δ*sco2730* Sc-M3 knockout mutant shows increased chromosomal end instability

*Streptomyces* chromosomes are inherently linear, featuring terminal inverted repeats and covalently bound terminal proteins. These linear chromosomes are intrinsically unstable and frequently undergo large deletions, particularly at their ends. When both telomeres are lost, the chromosome circularises to maintain viability [56]. As the *sco2730::Tn5062* (Sc-M1) mutant lacks chromosomal ends (Fig. 1j), we investigated chromosomal instability in the *sco2730/31* knockdown (Sc-M2) and the *Δsco2730* knockout (Sc-M3) mutants using the classical chloramphenicol-resistance assay. *S. coelicolor* is naturally resistant to chloramphenicol, and the gene conferring this resistance is located at the 3′ chromosomal end. Loss of chloramphenicol resistance therefore indicates deletion of the chromosomal end [52]. We observed the appearance of chloramphenicol-sensitive colonies at a frequency of approximately 0.5% in the Sc-M3 mutant, representing a tenfold increase compared with the Sc-M2 mutant and the *S. coelicolor* wild-type strain with and without the empty pNG4-SP44 overexpression plasmid [42] (Fig. 2c). These findings indicate that inactivation of the *sco2730* gene promotes chromosomal end loss.

### Construction of *S. coelicolor* and Δ*sco2730* strains lacking chromosomal ends (Sc-M4 and ScM5)

To investigate the possible effect of chromosomal end deletion on secondary metabolism, we constructed an *S. coelicolor* strain lacking chromosomal ends (Sc-M4). As detailed in the Methods section, we followed the approach developed by Volff et al. [57] to generate the chromosomal-end-removing plasmid pCER (Fig. 2d). This plasmid contains two DNA fragments corresponding to the 5′ and 3′ chromosomal ends, oriented to promote the deletion of terminal regions and the circularisation of the *S. coelicolor* linear chromosome via homologous recombination (Fig. 2e), thereby generating the Sc-M4 mutant (Fig. 2f). The 5′ and 3′ end regions selected correspond to those adjacent to the deletions identified in the Sc-M1 mutant, which are conserved across six *Streptomyces* model genomes in the StrepDB database (http://strepdb.streptomyces.org.uk/; *S. coelicolor*, *S. lividans*, *S. avermitilis*, *S. venezuelae*, *S. griseus* and *S. clavuligerus*). Chromosomal end deletion was confirmed by PCR (Fig. 2g) using chromosomal DNA from the Sc-M4 mutant and the *S. coelicolor* wild-type strain, together with the primers described in the Methods section. The resulting amplicons are depicted in Fig. 2f. The pCER plasmid was also used to delete the chromosomal ends in the Δ*sco2730* background, generating the Sc-M5 mutant.

### Metabolomic comparison of Sc-M3–M5 mutants shows that both the *sco2730/31* mutation and chromosomal-end deletion are required to reproduce Sc-M1 secondary metabolism activation

**Fig. 3:**
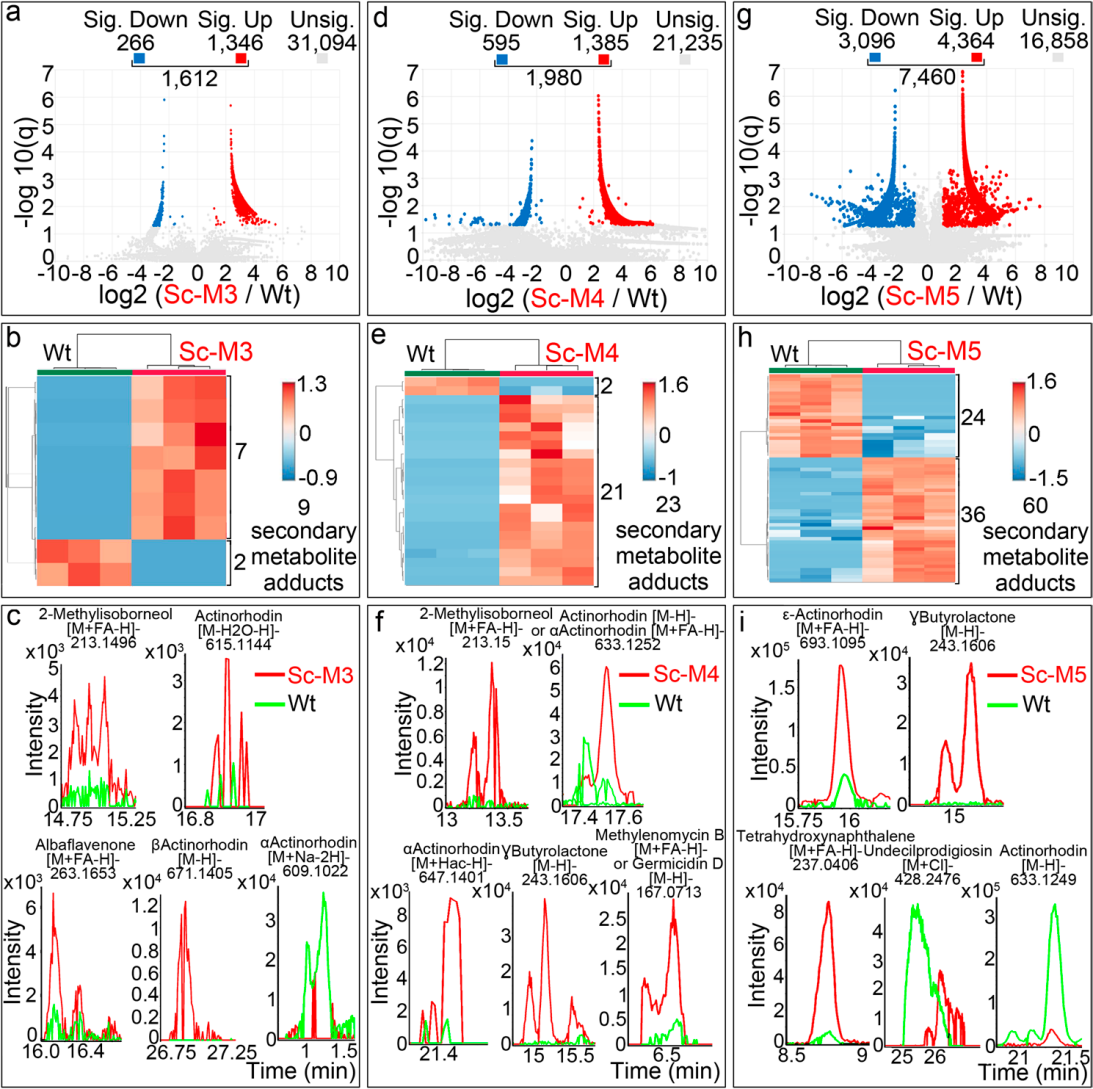
Differences in the metabolomes of the Δ*sco2730* mutant (Sc-M3), the chromosomal end-deletion mutant (Sc-M4), and the double mutant lacking both *sco2730* and chromosomal ends (Sc-M5) of *S. coelicolor*. **a**, **d**, **g** Volcano plots showing metabolites with significant (q-value ≤ 0.05 and |log₂ (mutant / *S. coelicolor* wild type)| ≥ ±1) and non-significant differences. **b**, **e**, **h** Heat maps illustrating the abundance of secondary-metabolite adducts showing significant differences across three biological replicates. **c**, **f**, **i** Extracted ion chromatograms showing representative secondary-metabolite adducts with significantly altered abundance between the mutants and the wild-type strain. Colours indicate: red, mutant; green, *S. coelicolor* wild type

To disentangle the respective contributions of *sco2730/31* inactivation and chromosomal-end loss to secondary metabolism, we independently examined their effects on the *S. coelicolor* metabolome by comparing the Sc-M3–M5 mutants with the wild-type strain (Fig. 3).

The Δ*sco2730* knockout Sc-M3 mutant, which affects the *sco2730/31* expression (Fig. 2a) [36] but retains its chromosomal ends, displayed significant changes in the abundance of 1,612 compounds (Fig. 3a), including nine secondary-metabolite adducts (Fig. 3b). Most of these adducts were more abundant in the mutant than in the wild type (Fig. 3b).

Similarly, the *S. coelicolor* strain lacking chromosomal ends (Sc-M4 mutant) showed alterations in 1,980 compounds (Fig. 3d), including 23 secondary-metabolite adducts (Fig. 3e), the majority of which also exhibited increased abundance relative to the wild-type strain.

The Δ*sco2730* mutant lacking chromosomal ends (Sc-M5) exhibited extensive metabolic remodelling, with significant differences in the abundance of 7,460 compounds (Fig. 3g), including 60 secondary-metabolite adducts (Fig. 3h), most of which were upregulated compared with the wild type.

Representative chromatograms of secondary-metabolite adducts showing significant differences in abundance between the mutants and the wild-type strain are shown in Figs. 3c, f, and i (chromatograms of significantly altered antibiotic adducts are presented in Additional File 5). In most cases, adduct abundance increased in the mutants (red traces in Figs. 3c, f, and i) relative to the wild type (green traces in Figs. 3c, f, and i).

**Fig. 4:**
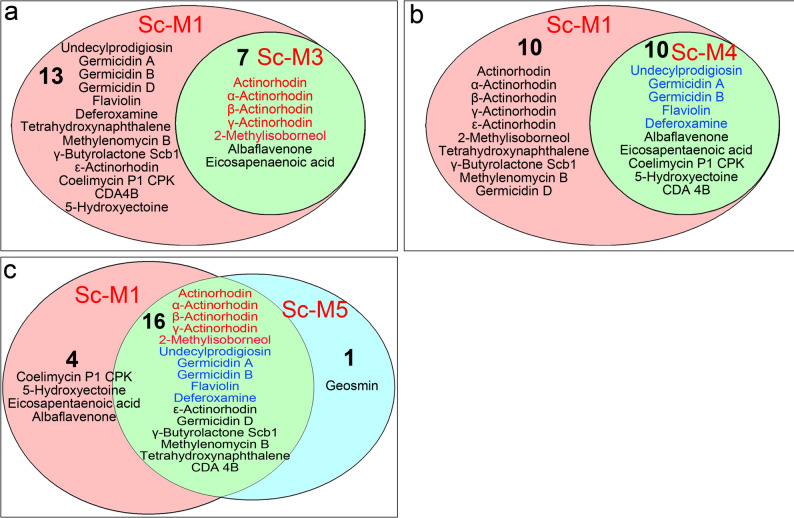
Venn diagrams illustrating secondary metabolites exhibiting adducts with differential abundance between the Sc-M3–M5 mutants and the *S. coelicolor* wild-type strain that are shared with the Sc-M1 mutant. **a** Sc-M1 vs. Sc-M3. **b** Sc-M1 vs. Sc-M4. **c** Sc-M1 vs. Sc-M5. Secondary metabolites whose production is affected in the Δ*sco2730* mutants, with or without chromosomal ends (Sc-M3 and Sc-M5), are labelled in red. Secondary metabolites whose production is altered in mutants lacking chromosomal ends (Sc-M4 and Sc-M5) are labelled in blue

We identified and quantified adducts from 21 secondary metabolites whose abundances differed significantly from those of the wild type in at least one of the five mutants analysed (Fig. 4). These adducts correspond to 16 distinct secondary metabolite biosynthetic pathways, including four actinorhodin variants (α, β, ε, and γ) and three germicidins (A, B, and D) (Fig. 4). Inactivation of *sco2730* (Sc-M3) and loss of the chromosomal ends (Sc-M4) each affected approximately half (seven and ten, respectively; Fig. 4a, b) of the 20 secondary metabolites altered in the Sc-M1 mutant. Only the combined *sco2730* inactivation and chromosomal-end loss in the Sc-M5 mutant reproduced the extensive activation of secondary metabolism observed in the Sc-M1 mutant, affecting 16 of the 20 secondary metabolites altered in the Sc-M1 mutant, as well as geosmin, which remained unchanged in Sc-M1 (Fig. 4c). Specifically, the production of actinorhodin, α-actinorhodin, β-actinorhodin, γ-actinorhodin, and 2-methylisoborneol (labelled in red in Fig. 4) was affected in both Δ*sco2730* mutants, with or without chromosomal ends (Sc-M3 and Sc-M5), whereas the production of undecylprodigiosin, germicidins A and B, flaviolin, and deferoxamine (labelled in blue in Fig. 4) was altered in the mutants lacking chromosomal ends (Sc-M4 and Sc-M5). As summarised in Fig. 4, metabolites labelled in red were consistently associated with the Δ*sco2730* mutation, whereas those labelled in blue were predominantly linked to chromosomal end deletion. These observations indicate that the *sco2730/31* copper chaperone–transporter system and the chromosomal ends independently modulate distinct subsets of secondary metabolite biosynthetic pathways, and that the combination of both mutations functions synergistically to amplify the activation of secondary metabolism.

### The Sc-M3-M5 mutants exhibit altered growth, spore germination, and copper accumulation in spores

**Fig. 5:**
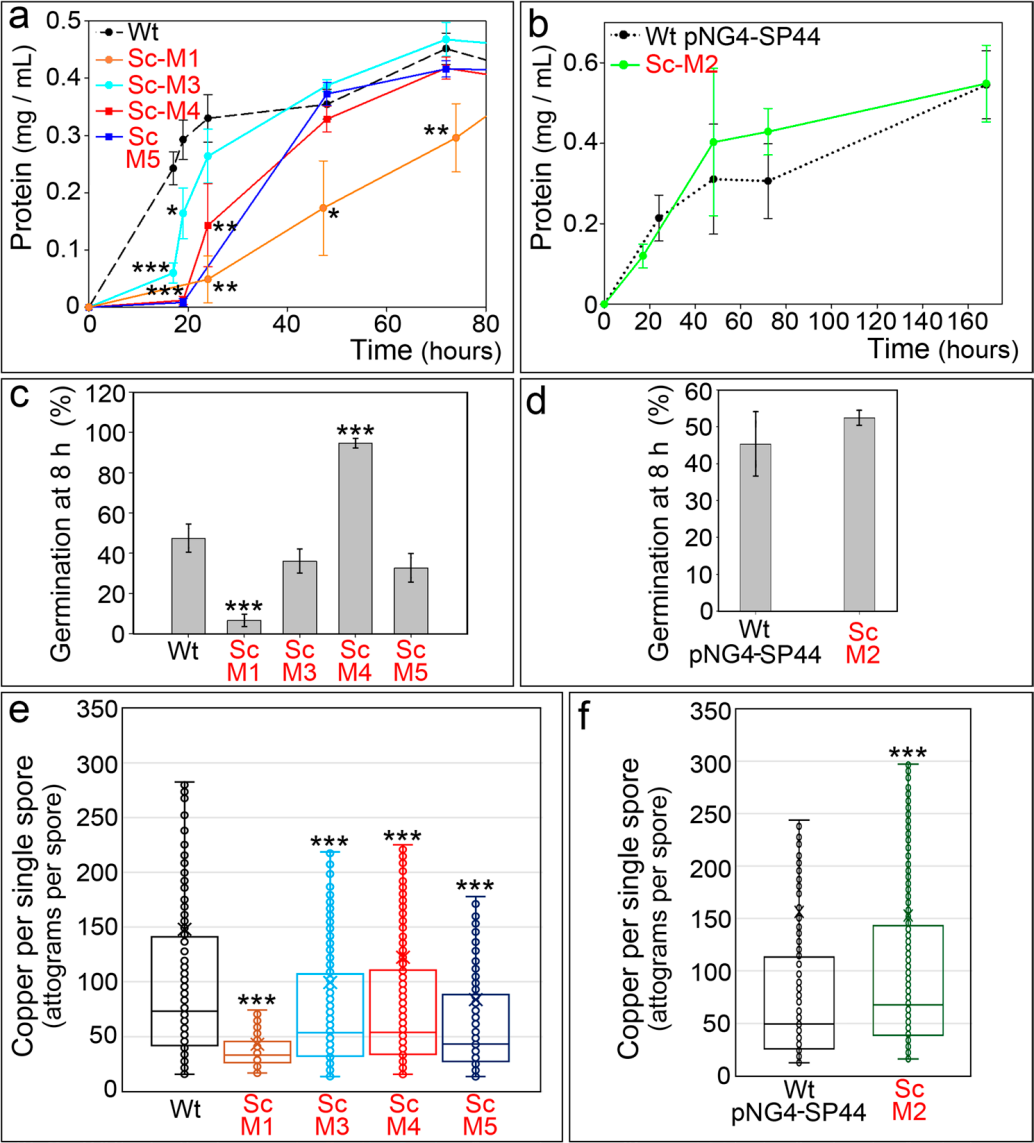
Growth, germination, and spore-associated copper content in the Sc-M1–M5 mutants. **a** Growth curves (mg protein per mL) of the Sc-M1, Sc-M3, Sc-M4, and Sc-M5 mutants compared with the *S. coelicolor* wild-type strain. **b** Growth curves (mg protein per mL) of the Sc-M2 mutant compared with the *S. coelicolor* wild-type strain carrying the empty pNG4-SP44 plasmid. **c** Germination percentage of the Sc-M1, Sc-M3, Sc-M4, and Sc-M5 mutants relative to the wild type. **d** Germination percentage of the Sc-M2 mutant relative to the wild type carrying the empty pNG4-SP44 plasmid. **e** Copper content per single spore in the Sc-M1, Sc-M3, Sc-M4, and Sc-M5 mutants compared with the wild type. (**f**) Copper content per single spore in the Sc-M2 mutant compared with the wild type carrying the empty pNG4-SP44 plasmid. Error bars in panels **a**–**d** indicate standard deviations from three biological replicates. Box-and-whisker plots in panels (**e**, **f**): centreline, median; box limits, upper and lower quartiles; whiskers, 1.5× the interquartile range. Asterisks denote significant differences relative to the corresponding wild-type control: *p < 0.05, **p < 0.01, ***p < 0.001

In addition to secondary metabolism activation, other notable phenotypes of the Sc-M1 mutant included delayed growth and spore germination, as well as an alteration in cytosolic copper transport, which was reflected by the accumulation of cytosolic copper in young vegetative hyphae [36]. We investigated whether these traits were also altered in the Sc-M1–M5 mutants (Fig. 5).

Growth was significantly reduced at early developmental time points in the Sc-M3–M5 mutants; however, this reduction was less pronounced than in the Sc-M1 mutant (Fig. 5a). The growth of the Sc-M2 mutant did not differ significantly from that of the wild-type strain carrying the empty expression plasmid pNG4-SP44 (Fig. 5b). In addition to the Sc-M1 mutant, spore germination was affected only in the Sc-M4 mutant (Fig. 5c, d). Cytosolic copper had previously been reported to be altered in the young hyphae of the Sc-M1 mutant [36]. Here, we analysed cytosolic copper in single spores of the Sc-M1–M5 mutants in comparison with the wild-type strain. Copper content per spore was significantly reduced in the Sc-M1 and Sc-M3–M5 mutants compared with the wild-type strain (Fig. 5e), whereas it was increased in Sc-M2 (Fig. 5f). Overall, growth, germination and spore copper content were differentially affected among the five mutants analysed. Reductions in growth and germination correlated with increasing copper accumulation in spores.

### Knockdown of the *sco2730/31* orthologues and chromosomal-end deletion synergistically affect secondary metabolism in *Streptomyces venezuelae*

**Fig. 6:**
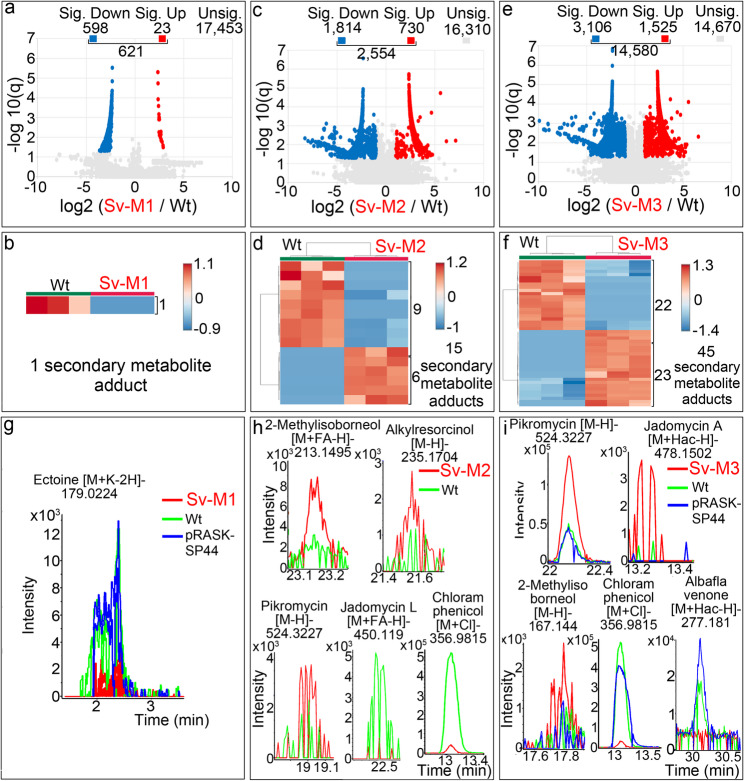
Differences in the metabolomes of the *S. venezuelae sco2730/31* knockdown mutant (Sv-M1), the chromosomal end-deletion mutant (Sv-M2), and the combined mutant (Sv-M3)**.** **a**, **c**, **e** Volcano plots showing metabolites with significant (q-value ≤ 0.05 and log₂ (mutant / *S. venezuelae* wild type) ≥ 1) and non-significant differences. **b**, **d**, **f** Heat maps illustrating the abundance of secondary-metabolite adducts that differ significantly across three biological replicates. **g**, **h**, **i** Extracted ion chromatograms of representative secondary-metabolite adducts with significantly altered abundance between each mutant and the wild-type strain. Colours indicate: red, mutant; green, *S. venezuelae* wild type; blue, *S. venezuelae* carrying the empty pRASK-SP44 plasmid

In a previous study, we tested the effect of *sco2730/31* consensus antisense RNA overexpression in *Streptomyces venezuelae* (Sv-M1 mutant) using the hygromycin-resistance plasmid pNG4-SP44 and observed only a modest impact on secondary metabolite production [42]. This finding was corroborated in the present work using the same antisense RNA, but expressed from a different vector, pRASK-SP44, which confers apramycin and kanamycin resistance and is compatible with the hygromycin resistance marker of the pCER plasmid used here for chromosomal end deletion. A total of 621 compounds showed significant differences between the Sv-M1 mutant and the wild-type strain (Fig. 6a), of which only one corresponded to a secondary metabolite adduct: ectoine, which was down-regulated in Sv-M1 (Fig. 6b) (Additional File 4).

The chromosomal ends of *S. venezuelae* were deleted using the pCER plasmid (Fig. 2d) to generate the Sv-M2 mutant. This mutation significantly altered the abundance of 2,554 compounds compared with the *S. venezuelae* wild-type strain (Fig. 6c), of which 15 corresponded to secondary-metabolite adducts (Fig. 6d) (Additional File 4).

When both mutations, *sco2730/31* orthologue down-regulation and chromosomal-end deletion, were combined in the same strain (Sv-M3 mutant), the abundance of 4,580 compounds was significantly altered compared with the wild-type strain (Fig. 6e), of which 45 corresponded to secondary-metabolite adducts (Fig. 6f) (Additional File 4).

Representative chromatograms of secondary-metabolite adducts showing significantly altered abundance between the mutants and the wild-type strain are presented in Fig. 6g–i (chromatograms of significantly altered antibiotic adducts are provided in Additional File 6). Although the differences in adduct abundance between the mutants and the wild type were statistically significant, the chromatograms indicate low production levels for most of them (within the e³ intensity range, resulting in chromatograms with irregular peak shapes) (Fig. 6g–i). The exceptions were the chloramphenicol, albaflavenone and pikromycin adducts, which exhibited higher intensities (within the e⁴–e⁵ range, resulting in chromatograms with sharp, well-defined peaks) (Fig. 6g–i). Chloramphenicol levels were markedly reduced in the Sv-M2 and Sv-M3 mutants (Fig. 6h, i), and albaflavenone production was strongly diminished in Sv-M3 (Fig. 6i). By contrast, pikromycin, a macrolide structurally related to erythromycin that has been reported in *S. venezuelae*, albeit at very low levels [21, 58], was markedly increased in the Sv-M3 mutant (Fig. 6i). None of these antibiotic adducts were affected by the presence of the empty pRASK-SP44 expression plasmid in the Sv-M1 and Sv-M3 mutants, as their abundance in *S. venezuelae* strains with and without the plasmid was comparable (blue and green traces in Fig. 6g and i; note that the Sv-M2 mutant does not harbour any plasmid).

## Discussion

Activation of *Streptomyces* BGCs requires an integrated combination of genetic, physiological, and biochemical approaches. In this study, we focused on triggering secondary metabolism through the modulation of copper homeostasis. Copper has been reported to pleiotropically regulate both hyphal differentiation and antibiotic production in *Streptomyces* [34–39]. Disruption of the *S. coelicolor* copper chaperone gene *sco2730* (Sc-M1 mutant), which also affects the adjacent *sco2731* gene encoding a P-type ATPase copper exporter, markedly enhances secondary metabolism [36]. Our aim was to identify the mutations responsible for the activation of secondary metabolism in the Sc-M1 mutant and to reproduce this activation in other *Streptomyces* species.

Since generating *sco2730* disruption mutants in multiple strains, such as those required for high-throughput screening campaigns, is not easily scalable, we previously developed an antisense mRNA construct targeting a conserved consensus sequence shared by *sco2730* and *sco2731* [42]. Although this construct enhanced secondary metabolism in *S. coelicolor* (Sc-M2 mutant), *S. venezuelae*, and *S. albidoflavus*, the effect was notably weaker (approximately half in the case of *S. coelicolor*) compared with the original Sc-M1 mutant [42]. Here, we investigated the differences between Sc-M1 and Sc-M2 mutants and discovered that disruption of *sco2730/31* increases the frequency of spontaneous chromosomal end deletion (Fig. 2c). This observation raised two questions: (i) whether the activation of secondary metabolism in Sc-M1 depends on both *sco2730/31* inactivation and chromosomal end deletion; and (ii) whether this activation can be reproduced in other *Streptomyces* species.

To address the first question, we analysed the individual and combined effects of *sco2730/31* mutation and chromosomal end loss using the Sc-M3, Sc-M4, and Sc-M5 mutants. All three mutants exhibited altered metabolomic profiles. The production of actinorhodin and its derivatives, as well as 2-methylisoborneol, appeared to depend primarily on *sco2730* inactivation and *sco2731* alteration (adducts labelled in red in Fig. 4). In contrast, the production of undecylprodigiosin, germicidins A/B, flaviolin, and deferoxamine was mainly affected by chromosomal end deletion (adducts labelled in blue in Fig. 4). Both *sco2730/31* inactivation and chromosomal end deletion independently enhanced secondary metabolism, but only their combination (Sc-M5) approached the strong activation observed in Sc-M1 [36], with up to 60 secondary-metabolite adducts altered across 17 distinct biosynthetic pathways (Figs. 3h and 4c). Transcriptional regulation of the *sco2730/31* region is complex, involving at least five distinct promoters [36] (Fig. 2a), which likely underlies the differences among the Sc-M3–M5 mutants, as each mutation affects these promoters differently. As in the Sc-M1 mutant, *sco2730/31* alteration also pleiotropically influenced other phenotypes, including growth, spore germination, and copper accumulation in spores, each differentially affected among the Sc-M3–M5 mutants (Fig. 5). Although the mutants exhibited early developmental delays in growth and spore germination, these differences were no longer apparent at later stages of growth, indicating that the mutations do not compromise their potential for large-scale or industrial applications. The milder phenotype observed in the Δ*sco2730* (Sc-M3) mutant compared with Sc-M1 may be explained by residual transcription of *sco2731*, likely driven by the downstream P5 promoter identified in this study. The transposon insertion in Sc-M1 may affect the transcriptional architecture of the *sco2730–2731* locus differently, potentially resulting in a distinct perturbation of *sco2731* expression relative to the Δ*sco2730* mutant.

To address the second question, whether secondary metabolism activation can be reproduced in other *Streptomyces* species, we tested the combined knockdown of the *S. venezuelae sco2730/31* orthologues using a *sco2730/31* consensus antisense RNA [42], together with chromosomal end deletion via the pCER plasmid constructed in this study (Fig. 2d). *S. venezuelae*, the species from which chloramphenicol was first discovered [59], responded similarly to *S. coelicolor*: the combined perturbation synergistically altered secondary metabolism in the Sv-M3 mutant (Fig. 6). The most affected antibiotics were chloramphenicol, which was markedly decreased (41-fold), and pikromycin, which increased 2.6-fold (Fig. 6i). Seventeen distinct pikromycin adducts were significantly elevated (Additional File 4). The pikromycin biosynthetic gene cluster (BGC) in *S. venezuelae* is largely silent under standard laboratory conditions, producing only low and inconsistent titres [58, 60]. Even targeted metabolic engineering has yielded only modest increases (2.2-fold compared with the wild type) [60], which is lower than the 2.6-fold enhancement observed here for the [M-H]⁻ pikromycin adduct (Fig. 6i). Additional silent pathways may also be affected; however, since metabolite identification relies on high-resolution mass spectrometry and theoretical exact masses, our dataset is inherently biased towards known, non-silent compounds. Of the 34 BGCs encoded in the *S. venezuelae* NRRL B-65,442 genome [61], exact theoretical masses could be matched for only 19, and of these, 11 were detected in our samples (Additional File 4).

Zhang et al. [62] recently reported that spontaneous chromosomal end loss occurs in a subpopulation of non-sporulating hyphae exhibiting diversified secondary metabolite profiles and increased antibiotic production, thereby providing protection to sporulating hyphae and conferring a colony-level ecological advantage. They proposed that such end-loss events represent an adaptive mechanism to safeguard the colony during sporulation-associated stress [62]. In our study, we show that chromosomal end deletion can be deliberately used to modulate secondary metabolism in *S. coelicolor* (Fig. 3) and *S. venezuelae* (Fig. 6). We further observed that chromosomal end loss is linked to copper handling during sporulation: the Sc-M4 mutant, which lacks chromosomal ends, accumulated less copper per spore than the wild type (Fig. 5e), and reduced copper transport in the Δ*sco2730* Sc-M3 mutant increased the frequency of chromosomal end-loss events (Fig. 2c). These observations suggest that chromosomal end loss, modulation of intracellular copper levels, and secondary metabolite production may have co-evolved to enhance antibiotic production and to protect sporulating hyphae.

The synergistic effect of *sco2730/31* inactivation and chromosomal end deletion on BGC activation likely reflects the interplay between genome architecture and global regulation. Inactivation of *sco2730/31* or its orthologues perturbs copper homeostasis, affecting redox balance, respiratory metabolism, specialised metabolism, and developmental progression [34–40]. Chromosomal end loss induces chromosome circularisation, altering DNA supercoiling, replication–transcription coupling, and higher-order chromosomal organisation, which may reshape global transcriptional programmes in *Streptomyces*, including the regulation of secondary metabolism [56, 57]. As chromosomal termini are enriched in accessory genes and some regulatory elements [63], their deletion may further rewire regulatory networks through changes in gene dosage and genomic context. Such structural and physiological shifts may influence pleiotropic regulators of development and antibiotic production. Beyond the phenotypic and metabolomic evidence presented here, further work is required to elucidate the molecular mechanisms linking copper-driven physiological reprogramming and large-scale chromosomal reorganisation to BGC activation. Although we observed that disruption of *sco2730/31* increases the frequency of chromosomal end loss in *S. coelicolor*, it remains to be determined whether disruption of their orthologues produces a similar effect in other streptomycetes, such as *S. venezuelae*, where comparable phenotypic assays based on chloramphenicol-resistance loss for detecting terminal deletions are currently not available.

## Conclusions

We demonstrate that simultaneous inactivation of the *sco2730/31* copper chaperone–transporter system and chromosomal end deletion synergistically alter secondary metabolism in *S. coelicolor* and *S. venezuelae*. Some secondary metabolite pathways were markedly upregulated, including previously silent pathways such as pikromycin in *S. venezuelae*, whereas others, such as chloramphenicol, were repressed. This work establishes the combined manipulation of *sco2730/31* orthologues and chromosomal end deletion as a broadly applicable approach for enhancing secondary metabolism across *Streptomyces* species, offering a new strategy for the challenging goal of activating silent biosynthetic pathways and discovering novel bioactive compounds.

## Supplementary Information

Below is the link to the electronic supplementary material.

Supplementary Material 1.

Supplementary Material 2.

Supplementary Material 3.

Supplementary Material 4.

Supplementary Material 5.

Supplementary Material 6.

## Data Availability

The authors declare that the data supporting the findings of this study is available within the article.
