# Supplementary material for: Inactivation of the sco2730/2731 copper chaperone–transporter system in Streptomyces coelicolor and its orthologs in Streptomyces venezuelae, together with chromosomal end deletion, greatly enhances secondary metabolism
Source: Microb Cell Fact. 2026 Apr 6;25:132. doi: 10.1186/s12934-026-03000-2 (PMC13214402; doi:10.1186/s12934-026-03000-2)
Supplement: Supplementary file 1 — Supplementary Material 1. [file 12934_2026_3000_MOESM1_ESM.pdf]

Additional File 1. *Streptomyces* and *Escherichia coli* strains used in this work.

Strain	Description	Reference
S.coelicolor M145	SCP1- SCP2- , reference strain	[1]
Streptomyces venezuelae NRRL B-65442	NRRL B-65442 strain	[1]
E. coli DH5α	F-Φ80lacZΔM15 Δ(lacZYA-argF)U169 recA1 endA1 hsdR17 (rk-, mk+) poa supE44 thi-1 gyrA96 relA1λ-	Invitrogen
E. coli ET12567/pUZ8002	*E. coli* ET12567 harbouring pUZ8002, a not self-transmissible plasmid which can mobilize *oriT*-containing plasmids by conjugation.	[2]
Sc-M1 mutant	*sco2730::Tn5062* mutant in *S.coelicolor* was generated by Tn5062 transposon insertion by conjugation with *E. coli* ET12567/pUZ8002	[3]
Sc-M2 mutant	*sco2730/31* antisense mRNA in *S. coelicolor*. $Hygro^R$	[4]
Sc-M3 mutant	*sco2730* knockout in *S. coelicolor*. $Apra^R$	This study
Sc-M4 mutant	*S. coelicolor* without chromosomal ends. $Hygro^R$	This study
SC-M5 mutant	*S. coelicolor* combined a *SCO2730* knockout with the deletion of the chromosomal ends. $Apra^R Kan^R Hygro^R$	This study
Sv-M1 mutant	*sco2730/31* antisense mRNA in *S. venezuelae*. $Apra^R$ $Kana^R$	This study
Sv-M2 mutat	*S. venezuelae* without chromosomal ends. $Hygro^R$	This study
Sv-M3 mutant	*S. venezuelae* combined the sco2730/31 antisense mRNA with the deletion of the chromosomal ends. $Apra^R Kan^R Hygro^R$	This study

References

1. Kieser T: *Practical Streptomyces genetics*. Norwich: John Innes Foundation; 2000.
2. Flett F, Mersinias V, Smith CP: **High efficiency intergeneric conjugal transfer of plasmid DNA from *Escherichia coli* to methyl DNA-restricting streptomycetes.** *FEMS Microbiol Lett* 1997, **155:**223-229.
3. Gonzalez-Quinonez N, Corte-Rodriguez M, Alvarez-Fernández-Garcia R, Rioseras B, Lopez-Garcia MT, Fernández-Garcia G, Montes-Bayon M, Manteca A, Yagüe P: **Cytosolic copper is a major modulator of germination, development and secondary metabolism in *Streptomyces coelicolor*** *Sci Rep* 2019, **9:**4214.
4. González-Quiñónez N, Gutiérrez-Del-Río I, García-Cancela P, Fernández-García G, Alonso-Fernández S, Yagüe P, Pérez-Valero Á, Montes-Bayón M, Lombó F, Manteca Á: **The Modulation of SCO2730/31 Copper Chaperone/Transporter Orthologue Expression Enhances Secondary Metabolism in Streptomycetes.** *International Journal of Molecular Sciences* 2021, **22:**10143.
