# Supplementary material for: Inactivation of the sco2730/2731 copper chaperone–transporter system in Streptomyces coelicolor and its orthologs in Streptomyces venezuelae, together with chromosomal end deletion, greatly enhances secondary metabolism
Source: Microb Cell Fact. 2026 Apr 6;25:132. doi: 10.1186/s12934-026-03000-2 (PMC13214402; doi:10.1186/s12934-026-03000-2)
Supplement: Supplementary file 2 — Supplementary Material 2. [file 12934_2026_3000_MOESM2_ESM.pdf]

Additional File 2. Primers used in this work.

Primer	Sequence (5'→3')	Reference
M13F	GTAAAACGACGGCCAG	Invitrogen
M13R	CAGGAAACAGCTATGAC	Invitrogen
oriTFa	ACATGTAGGTCGACGGATCTTTTCCG	This study
oriTRa	GTTAACATCATCTCCTCCAGGGTGGT	This study
oriTPciI	CGCCAGCAACGCGGC	This study
oriTFb	GCCTGGACGTGTACG	This study
oriTRb	TTTCTACGGGGTCTGACGCT	This study
oriT2730	TCGTACGCCCCGGCGAGAA	This study
Tn2730F	GGGAGTTGGCGCTCGTG	This study
Tn2730R	GGGGACGAAGATCCCCGA	This study
hygroF	ACTAGTGGTACCAGTGAGCGTTTTTCAACCT	This study
hygroR	GATATCAAAGACAATCCCCGATCCGCTC	This study
5primaF	GGATCCTCCACCCCGGCGGTGCC	This study
5primaR	ACTAGTGTGCGGCAAGGGGGATTGGT	This study
3primaF	GATATCGCGGGGTGCCCATGGCGA	This study
3primaR	GCGGCCGCCGCGTCCCCGAAGAGCCG	This study
TOPOHindIII	TCACACAGGAAACAGCTATGACCAT	This study
5pSpeI	GATGTGCTCAGTATCAC	This study
110v3F1	CAAGCAAGGCCCATGTACCC	This study
110v3R1	GACCGATCTGATGTGCTCAGTA	This study
110v3R2	TGTCCAGAGTCGATCGGTAGG	This study
sven3566F	GTGTTGCCGTCCTGCTTCT	This study
sco3798intR	TCAGGTCCATGACGTTTCCC	[1]
circu5F	CGCAGGAACTCCGGGTTGA	This study
circu5R	AGGTTGAAAAACGCTCACTGGTA	This study
circu3F	ATTGCTGGGGCAACACGTGGA	This study
circu3R	AGCTTCCGGCAGGACCCCT	This study
P5F	GAAGAAGCTCAACCGCATGG	This study
P5R	CAGGTAGATCGCGGAGGTG	This study

References

1. Fernández-García G, González-Quiñónez N, Rioseras B, Alonso-Fernández S, Fernández J, Lombó F, Manteca Á: **The SCO2102 Protein Harbouring a DnaA II Protein-Interaction Domain Is Essential for the SCO2103 Methylenetetrahydrofolate Reductase Positioning at *Streptomyces* Sporulating Hyphae, Enhancing DNA Replication during Sporulation.** *International Journal of Molecular Sciences* 2022, **23:**4984.
