# Supplementary material for: Inactivation of the sco2730/2731 copper chaperone–transporter system in Streptomyces coelicolor and its orthologs in Streptomyces venezuelae, together with chromosomal end deletion, greatly enhances secondary metabolism
Source: Microb Cell Fact. 2026 Apr 6;25:132. doi: 10.1186/s12934-026-03000-2 (PMC13214402; doi:10.1186/s12934-026-03000-2)
Supplement: Supplementary file 5 — Supplementary Material 5. [file 12934_2026_3000_MOESM5_ESM.pdf]

Additional File 5. Extracted ion chromatograms showing significantly altered secondary metabolites in the *S. coelicolor* mutants compared with the wild-type strain. One adduct for each secondary metabolite exhibiting significant differences between the Sc-M1–M5 mutants and the wild-type strain is presented. (a) Sc-M1 and Sc-M2. (b) Sc-M3. (c) Sc-M4. (d) Sc-M5.

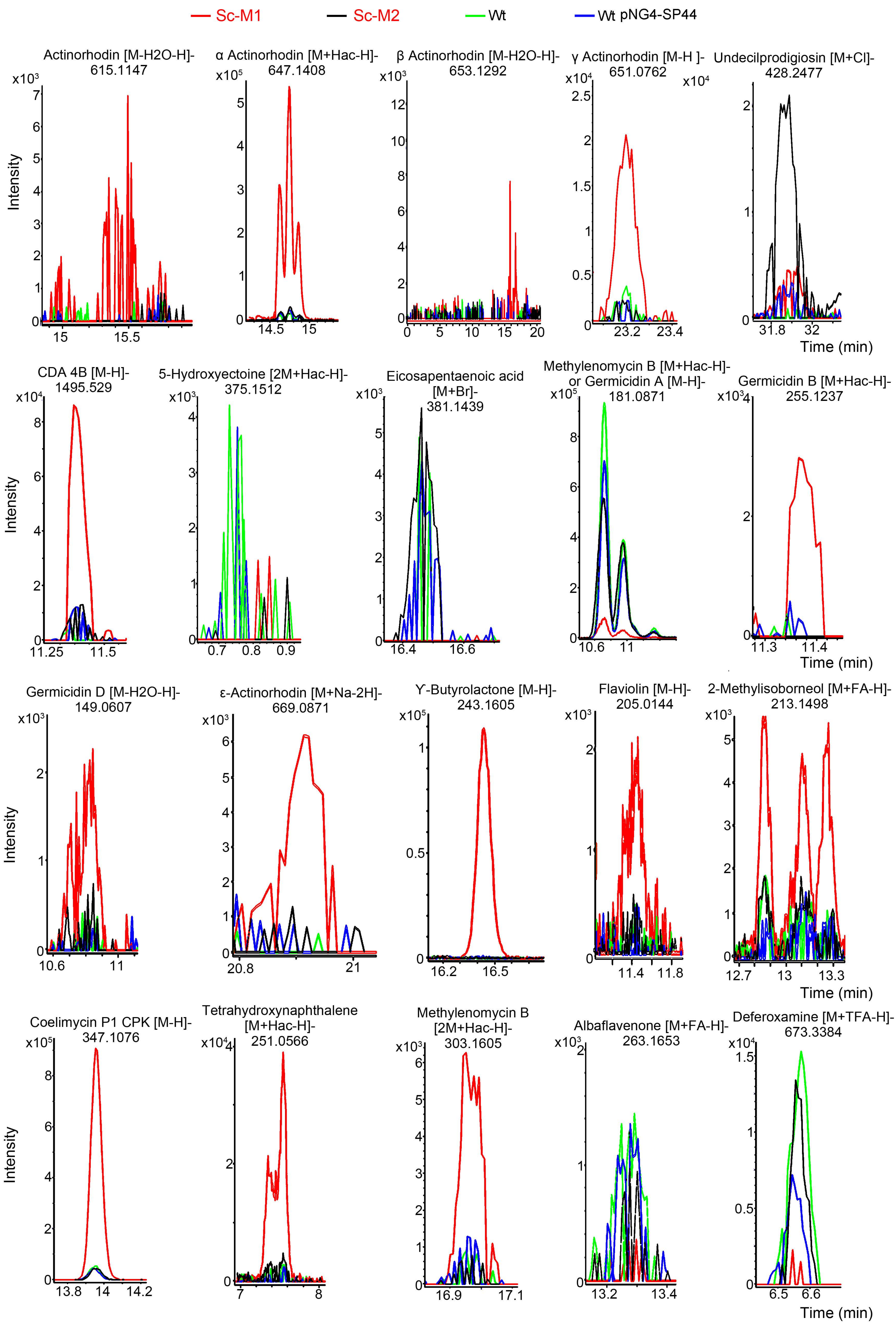

Sc-M1
Sc-M2
Wt
Wt pNG4-SP44
Actinorhodin [M-H2O-H]- 615.1147
α Actinorhodin [M+Hac-H]- 647.1408
β Actinorhodin [M-H2O-H]- 653.1292
γ Actinorhodin [M-H]- 651.0762
Undecilprodigiosin [M+Cl]- 428.2477
CDA 4B [M-H]- 1495.529
5-Hydroxyectoine [2M+Hac-H]- 375.1512
Eicosapentaenoic acid [M+Br]- 381.1439
Methylenomycin B [M+Hac-H]- or Germicidin A [M-H]- 181.0871
Germicidin B [M+Hac-H]- 255.1237
Germicidin D [M-H2O-H]- 149.0607
ε-Actinorhodin [M+Na-2H]- 669.0871
Y-Butyrolactone [M-H]- 243.1605
Flaviolin [M-H]- 205.0144
2-Methylisoborneol [M+FA-H]- 213.1498
Coelimycin P1 CPK [M-H]- 347.1076
Tetrahydroxynaphthalene [M+Hac-H]- 251.0566
Methylenomycin B [2M+Hac-H]- 303.1605
Albaflavenone [M+FA-H]- 263.1653
Deferoxamine [M+TFA-H]- 673.3384
Intensity
Time (min)

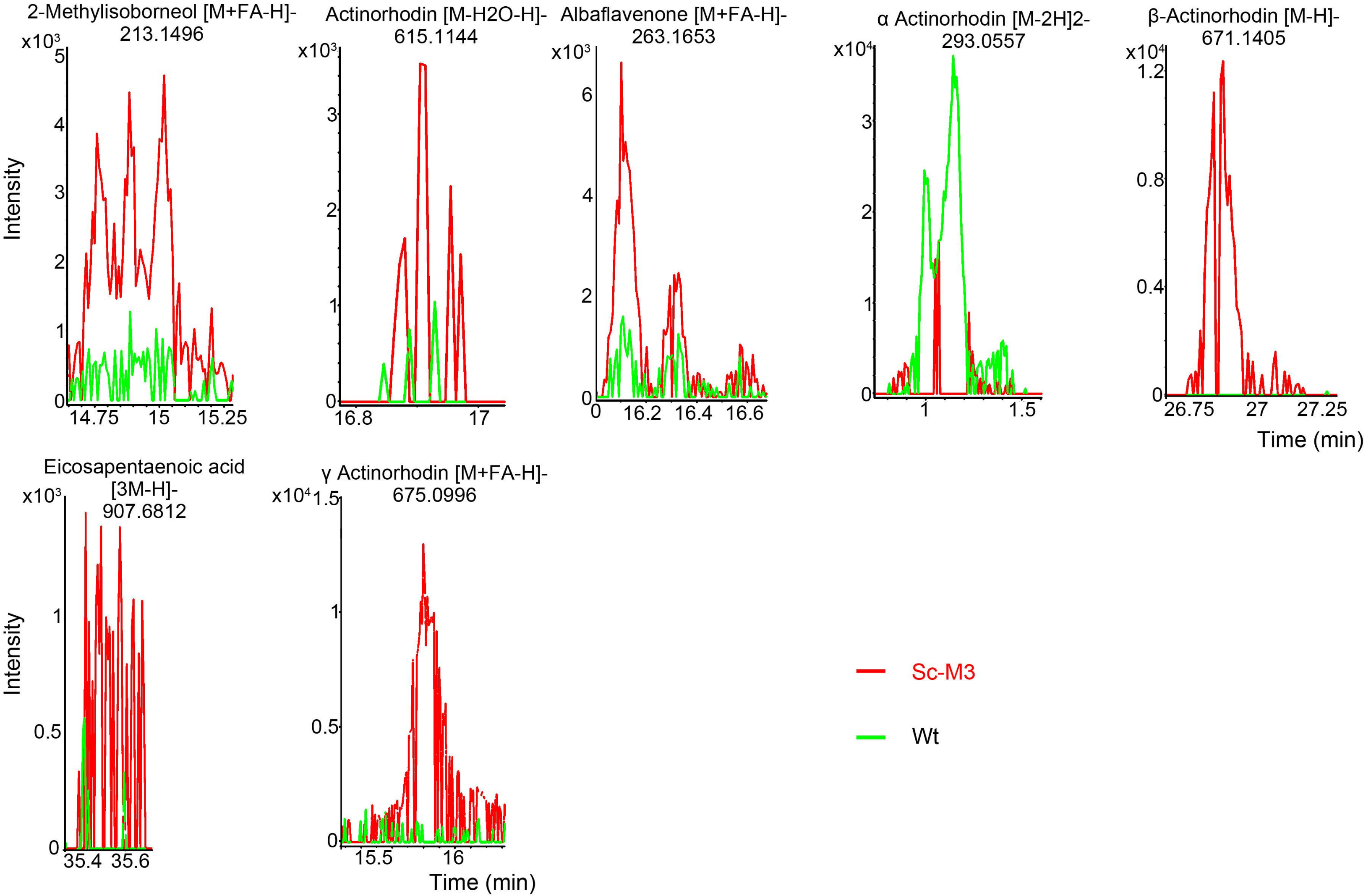
2-Methylisoborneol [M+FA-H]-
213.1496
Actinorhodin [M-H2O-H]-
615.1144
Albaflavenone [M+FA-H]-
263.1653
α Actinorhodin [M-2H]2-
293.0557
β-Actinorhodin [M-H]-
671.1405
Eicosapentaenoic acid
[3M-H]-
907.6812
γ Actinorhodin [M+FA-H]-
675.0996
Intensity
Time (min)
x10³
x10⁴
Sc-M3
Wt

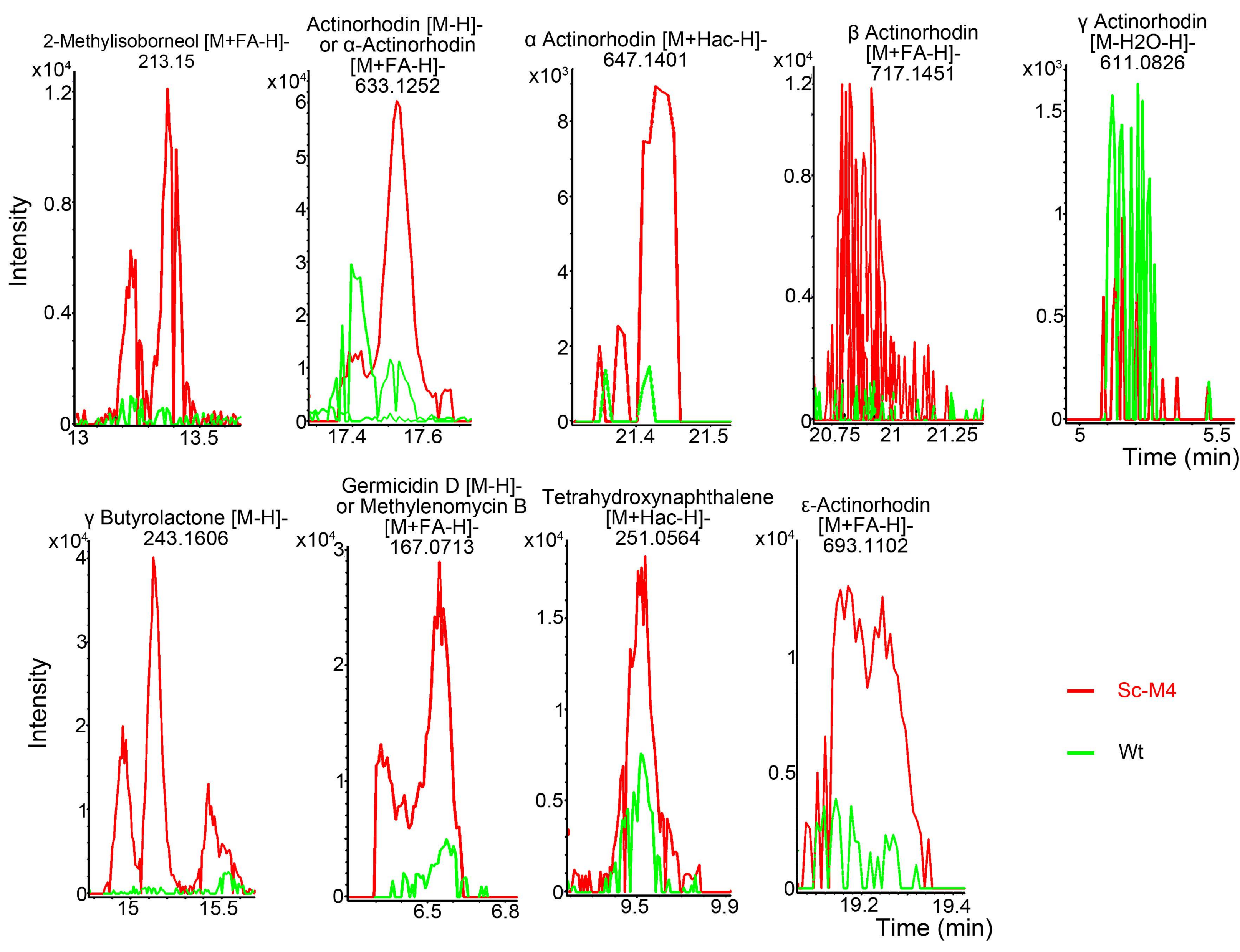
2-Methylisoborneol [M+FA-H]- 213.15
Actinorhodin [M-H]- or α-Actinorhodin [M+FA-H]- 633.1252
α Actinorhodin [M+Hac-H]- 647.1401
β Actinorhodin [M+FA-H]- 717.1451
γ Actinorhodin [M-H2O-H]- 611.0826
γ Butyrolactone [M-H]- 243.1606
Germicidin D [M-H]- or Methylenomycin B [M+FA-H]- 167.0713
Tetrahydroxynaphthalene [M+Hac-H]- 251.0564
ε-Actinorhodin [M+FA-H]- 693.1102
Intensity
Time (min)
Sc-M4
Wt

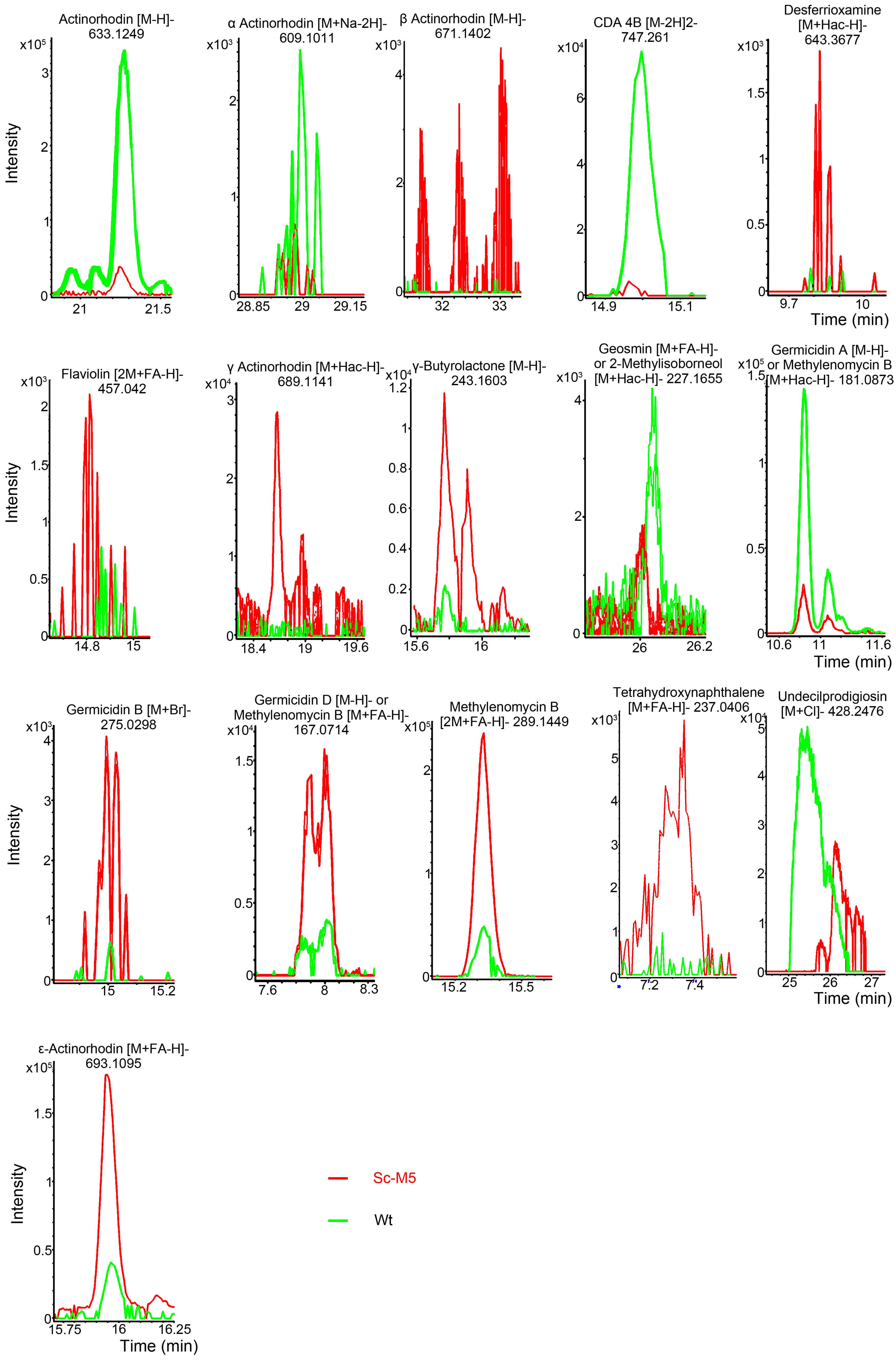
Actinorhodin [M-H]- 633.1249
α Actinorhodin [M+Na-2H]- 609.1011
β Actinorhodin [M-H]- 671.1402
CDA 4B [M-2H]2- 747.261
Desferrioxamine [M+Hac-H]- 643.3677
Flaviolin [2M+FA-H]- 457.042
γ Actinorhodin [M+Hac-H]- 689.1141
γ-Butyrolactone [M-H]- 243.1603
Geosmin [M+FA-H]- or 2-Methylisoborneol [M+Hac-H]- 227.1655
Germicidin A [M-H]- or Methylenomycin B [M+Hac-H]- 181.0873
Germicidin B [M+Br]- 275.0298
Germicidin D [M-H]- or Methylenomycin B [M+FA-H]- 167.0714
Methylenomycin B [2M+FA-H]- 289.1449
Tetrahydroxynaphthalene [M+FA-H]- 237.0406
Undecilprodigiosin [M+Cl]- 428.2476
ε-Actinorhodin [M+FA-H]- 693.1095
Intensity
Time (min)
Sc-M5
Wt
