# Supplementary material for: Inactivation of the sco2730/2731 copper chaperone–transporter system in Streptomyces coelicolor and its orthologs in Streptomyces venezuelae, together with chromosomal end deletion, greatly enhances secondary metabolism
Source: Microb Cell Fact. 2026 Apr 6;25:132. doi: 10.1186/s12934-026-03000-2 (PMC13214402; doi:10.1186/s12934-026-03000-2)
Supplement: Supplementary file 6 — Supplementary Material 6. [file 12934_2026_3000_MOESM6_ESM.pdf]

Additional File 6. Extracted ion chromatograms showing significantly altered secondary metabolites in the *S. venezuelae* mutants compared with the wild-type strain. One adduct for each secondary metabolite exhibiting significant differences between the Sv-M2–M3 mutants and the wild-type strain is presented. (a) Sv-M2. (b) Sv-M3.

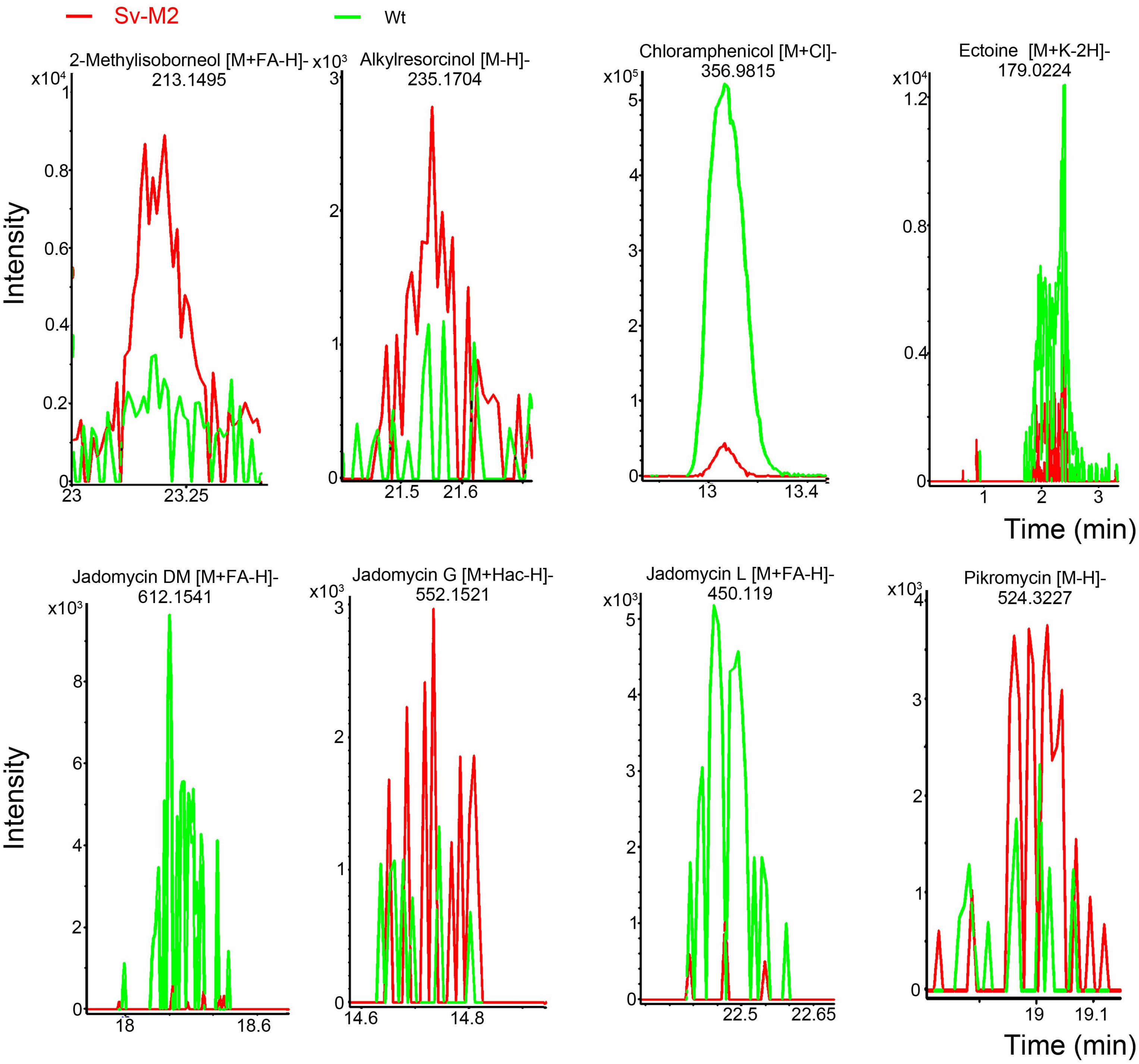

Sv-M2
Wt
Intensity
2-Methylisoborneol [M+FA-H]- 213.1495
Alkylresorcinol [M-H]- 235.1704
Chloramphenicol [M+Cl]- 356.9815
Ectoine [M+K-2H]- 179.0224
Jadomycin DM [M+FA-H]- 612.1541
Jadomycin G [M+Hac-H]- 552.1521
Jadomycin L [M+FA-H]- 450.119
Pikromycin [M-H]- 524.3227
Time (min)

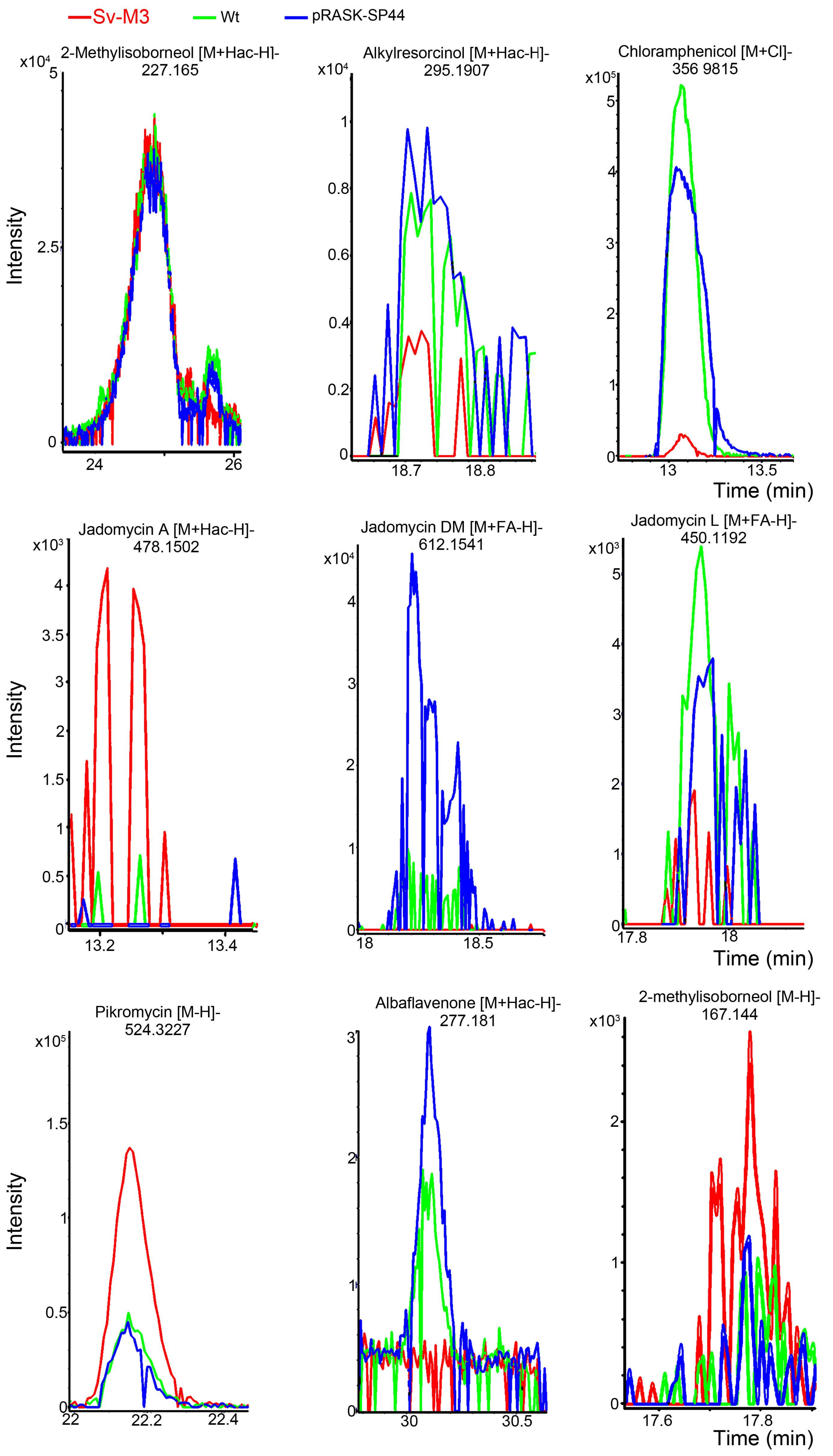
Sv-M3
Wt
pRASK-SP44
2-Methylisoborneol [M+Hac-H]-
227.165
Alkylresorcinol [M+Hac-H]-
295.1907
Chloramphenicol [M+Cl]-
356 9815
Jadomycin A [M+Hac-H]-
478.1502
Jadomycin DM [M+FA-H]-
612.1541
Jadomycin L [M+FA-H]-
450.1192
Pikromycin [M-H]-
524.3227
Albaflavenone [M+Hac-H]-
277.181
2-methylisoborneol [M-H]-
167.144
Intensity
Time (min)
x10⁴
x10⁵
x10³
